# Visualization of human karyopherin beta-1/importin beta-1 interactions with protein partners in mitotic cells by co-immunoprecipitation and proximity ligation assays

**DOI:** 10.1038/s41598-018-19351-9

**Published:** 2018-01-30

**Authors:** Laura Di Francesco, Annalisa Verrico, Italia Anna Asteriti, Paola Rovella, Pietro Cirigliano, Giulia Guarguaglini, Maria Eugenia Schininà, Patrizia Lavia

**Affiliations:** 1 Dipartimento di Scienze Biochimiche, Sapienza University of Rome, Piazzale Aldo Moro 5, 00185 Rome, Italy; 2 Institute of Molecular Biology and Pathology (IBPM), CNR National Research Council of Italy, Via degli Apuli 4, 00185 Rome, Italy; 3 Nikon Instruments S.p.A., Campi Bisenzio, Italy; 4 Present Address: Unit of Human Microbiome, Bambino Gesù Children’s Hospital-IRCCS, Rome, Italy

## Abstract

Karyopherin beta-1/Importin beta-1 is a conserved nuclear transport receptor, acting in protein nuclear import in interphase and as a global regulator of mitosis. These pleiotropic functions reflect its ability to interact with, and regulate, different pathways during the cell cycle, operating as a major effector of the GTPase RAN. Importin beta-1 is overexpressed in cancers characterized by high genetic instability, an observation that highlights the importance of identifying its partners in mitosis. Here we present the first comprehensive profile of importin beta-1 interactors from human mitotic cells. By combining co-immunoprecipitation and proteome-wide mass spectrometry analysis of synchronized cell extracts, we identified expected (e.g., RAN and SUMO pathway factors) and novel mitotic interactors of importin beta-1, many with RNA-binding ability, that had not been previously associated with importin beta-1. These data complement interactomic studies of interphase transport pathways. We further developed automated proximity ligation assay (PLA) protocols to validate selected interactors. We succeeded in obtaining spatial and temporal resolution of genuine importin beta-1 interactions, which were visualized and localized *in situ* in intact mitotic cells. Further developments of PLA protocols will be helpful to dissect importin beta-1-orchestrated pathways during mitosis.

## Introduction

Human karyopherin beta-1/importin beta-1 is a major effector of the GTPase RAN and a highly conserved member of the superfamily of nuclear transport receptors. As such, it regulates key cellular functions^1^, including i) protein import in interphase nuclei, underlying the fundamental processes of DNA replication, DNA repair, transcriptional and epigenetic control of gene expression^2,3^, ii) the localization and activity of factors implicated in mitotic spindle organization and function after nuclear envelope (NE) breakdown^4^, and, iii) the reorganization of the  NE, nuclear pore complexes (NPCs)  and nuclear structure at mitotic exit^5,6^.

Importin beta-1 has a well-characterized modular structure composed of HEAT repeats included in distinct functional domains^7^. Its interphase partners are both direct and indirect. Indirect partners include proteins carrying nuclear localization signals (NLS), recognized by an adaptor molecule belonging to the importin alpha subfamily. Using its C-terminal region, importin beta-1 interacts with importin alpha adaptor/NLS cargo, forming “classical” trimeric import complexes. In the nucleus, the GTPase RAN, loaded with GTP (RANGTP), binds importin beta-1 at its N-terminal region, thus destabilizing interactions at the C-terminus and triggering the cargo release from the import complex. Importin beta-1 also binds several proteins directly, including nucleoporins (NUPs) in their phenyl-glycine (FG)- and FxFG-rich regions, when crossing nuclear pores during nuclear import. Importin beta-1 also directly recognizes a variety of unrelated cargoes acting in different pathways^2,8^.

After NE breakdown, importin beta-1 takes on global regulatory roles in mitotic spindle organization and function^4,9,10^. In this mitotic mode, importin beta-1 generally inhibits factors with which it interacts^2^, and thus prevents their unscheduled activity and the premature onset of events during mitosis. Analogous to the mechanism operating in nuclear import, RANGTP binding to importin beta-1 releases mitotic “cargoes” in a free, biologically active form, thus enabling relevant mitotic event(s) to take place. When overexpressed in human cells, importin beta-1 overrides RANGTP regulation and keeps its mitotic partners in an inhibited state: this causes complex mitotic abnormalities, even under conditions under which nuclear import is not overtly affected^11–14^, indicating that mitosis is most sensitive to altered importin beta-1 levels. Regulated expression of importin beta-1 is therefore critical in control of cell division^4^. Importin beta-1 is overexpressed in many cancer types that generally display high genomic instability, e.g. cervical^15^ and gastric^16^ carcinoma, as well as diffuse large B-cell lymphoma^17^. Because many cancer types are dependent on importin beta-1 expression for their proliferation and survival^15–20^, importin beta-1 has been proposed as a novel therapeutic target^21^ and inhibitors are being developed, e.g. Importazole^22^ and INI-43^23^.

Interactomic analyses of importin-1 beta have been developed in interphase cultures to get insight into mechanisms of transport, based on stable isotope labeling by amino acids in cell cultures (SILAC)^24^, and, more recently, in a comparative study of twelve human importin family members to depict specificities in recognition of cargoes in distinct import pathways^25^. Studies of importin beta-1 mitotic interactors are less complete. In an early analysis of human importin beta-1 mitotic partners, we used MALDI-TOF mass spectrometry (MS) to examine importin beta-1 co-immunoprecipitates from mitotic-enriched cell cultures after thymidine arrest and release: we identified NUP358/ RAN-binding protein 2 (RANBP2), a large NUP with RAN-binding domains and E3 ligase activity for small ubiquitin-like modifier (SUMO) peptides, and RAN GTPase-activating protein 1 (RANGAP1) in the SUMO-conjugated form (RANGAP1-SUMO1)^14^. RANBP2 and RANGAP1-SUMO1 themselves form a stable complex throughout the cell cycle^26^. The complex (indicated as RRSU, for RANBP2/RANGAP1-SUMO1/UBC9) includes the SUMO E2 conjugating enzyme UBC9 and has enhanced SUMO ligase activity^27^. RANBP2 and RANGAP1-SUMO1 localize at the spindle microtubules (MTs) and accumulate to kinetochores (KTs) after MT attachment^28^. Importin beta-1 also localizes to the mitotic spindle MTs and poles^12^, and its interaction with RANBP2 prevents the RRSU premature localization at KTs prior to MT attachment^14,29^. Recent work has shown that this is essential to regulate the timing of SUMO conjugation of KT factors, and hence KT functions^30,31^. However, beyond individual studies of specific mitotic factors, a comprehensive view of importin beta-1 interactors in mitotic cells is currently lacking.

Here we present a comprehensive identification of importin beta-1 interactors from homogeneously synchronized mitotic cells using co-immunoprecipitation (co-IP) experiments hyphenated to a high resolution MS analysis. We identify expected partners, e.g. members of the RAN and SUMO pathways, as well as interactors that had not been previously associated with importin beta-1. Most mitotic protein interactions are highly dynamic in time and in space, and innovative methodologies are being developed to unravel specificities and variations. *In situ* proximity ligation assays (is-PLA) is an informative detection technique to visualize protein interactions at their intracellular site^32^. We have developed an automated PLA protocol to validate selected importin beta-1 interactions: that has enabled us to visualize the interactions of interest during stages of mitotic progression. Coupling the interactome analysis with automated is-PLA imaging provides valuable information on processes through which importin beta-1 can operate in mitosis.

## Results

### Co-immunoprecipitation of importin beta-1 and its partners from synchronized mitotic cells

**Figure 1:**
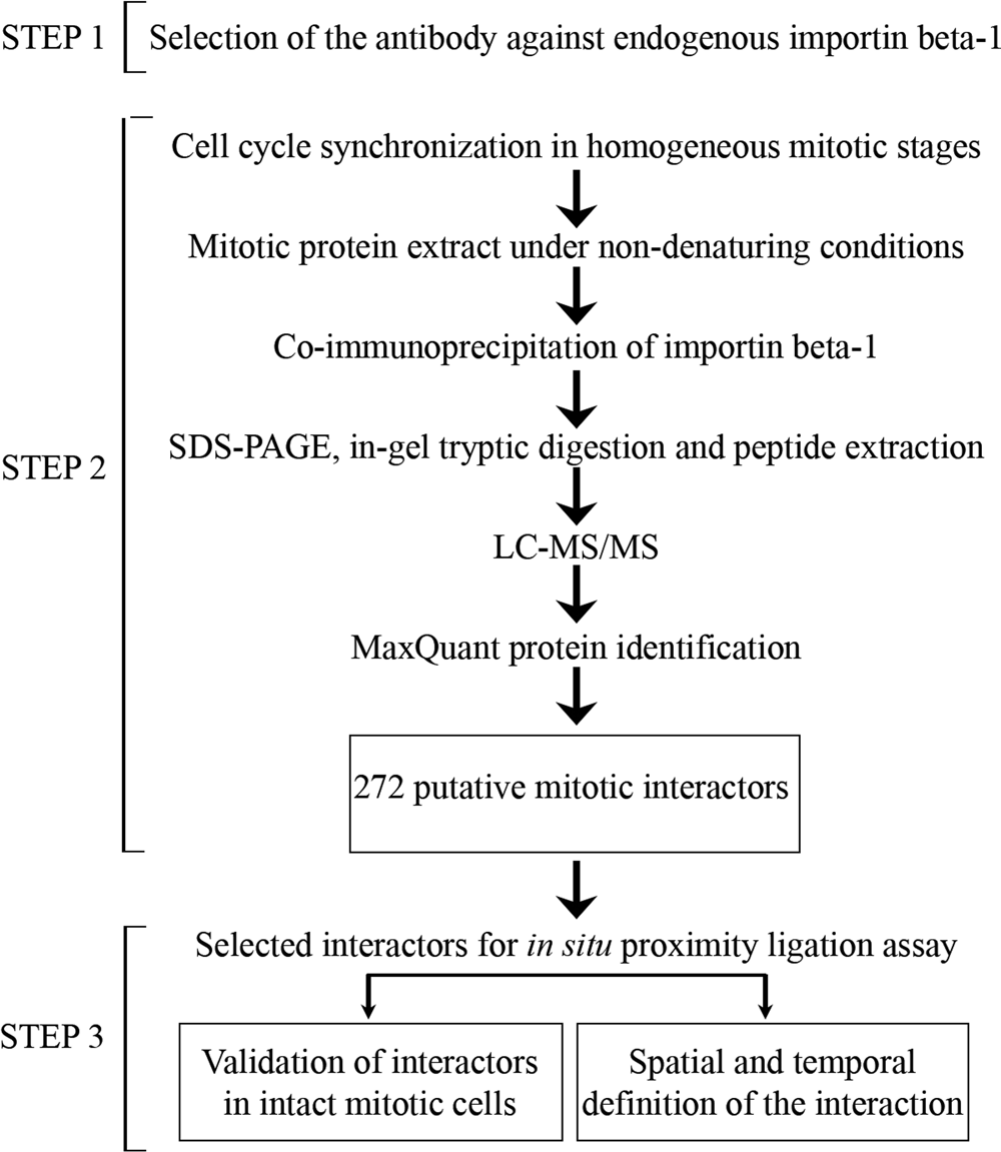
Schematics of the three-step interactomic workflow developed in this work. After selection of the importin beta-1 antibody (step 1), co-immunoprecipitating mitotic partners are identified (non denaturing conditions, low-stringency) and profiled in step 2; in step 3, selected partners are then validated by an automated is-PLA protocol.

To identify the interactions of importin beta-1 with protein partners during mitosis, we developed the workflow schematized in Fig. 1. Briefly, after an antibody selection step, large scale immunoprecipitates of importin beta-1 complexes from mitotic HeLa cells were analyzed by mass spectrometry (Step 2) and selected interactors were validated by is-PLA (Step 3).

Prior to developing affinity purification and mass spectrometry (AP/MS)^33^ analyses, we had to address two preliminary methodological issues, concerning the importin-beta-1 antibody bait and the purity of mitotic cell populations. Because importin beta-1 can engage various domains to interact with partners, different antibodies might be differentially hindered in co-IP experiments by importin beta-1 partners with which they may compete. We tested commercial antibodies against full-length or specific epitopes of importin beta-1 (map in Supplementary Fig. 1a). To compare with previous work^14^, protein extracts were prepared from thymidine-arrested (G1/S transition) and released cell cultures collected at mitotic round-up using the “shake-off” procedure. After cross-linking to beads and incubation with equal extract amounts, the antibodies showed differential effectiveness in their yield of immunoprecipitated importin beta-1 (Supplementary Fig. 1b). We compared the co-IP obtained with the three most effective antibodies (2, 4 and 5 in Supplementary Fig. 1a) by low-scale MALDI-TOF analysis. Antibodies against the full-length protein, 4 and 5, consistently identified RAN and RANBP2 in the importin beta-1 co-IP, as expected (Supplementary Fig. 1c). Antibody 5 proved particularly effective in the IP yield (Supplementary Fig. 1b) and was used thereafter.

Preliminarily we also needed to ensure homogeneity of mitotic synchronization. We realized that thymidine-arrested and released cultures tended to loose synchrony during release into S and G2 phases, reaching mitotic stages somewhat asynchronously (Supplementary Fig. 2a). We therefore compared protocols that synchronize the cell cycle closer to mitosis, using: i) the CDK1 inhibitor RO3306 (arrest at G2/M boundary), ii) the monastrol derivative STLC^34^, which inhibits the kinesin Eg5 and arrests cells in late G2/early prometaphase with unseparated centrosomes. By immunofluorescence (IF), STLC-arrested and released cell cultures, though being more synchronous compared to thymidine release, included many mitoses still exhibiting monopolar spindles 1 hour after wash-out (Supplementary Fig. 2c), suggesting that released cells progress through mitosis before they fully recover from Eg5 inhibition. RO3306-induced arrest and release (30–60 minutes) proved both effective and reversible, yielding cells that accomplished mitosis proficiently and synchronously (Supplementary Fig. 2b). We conclude that RO3306-mediated synchronization and release is suitable for large-scale proteomics in mitotic cell populations.

### Profiling importin beta-1 mitotic interactors

In order to profile protein partners contacted by importin beta-1 in mitosis, we pulled down importin beta-1 from HeLa cells under non-denaturing conditions. Mitotic cell extracts were prepared from RO3306-synchronized and released cells collected by mechanical shake-off at mitotic round-up, then incubated with importin beta-1 antibody for preparative co-IP under low salt stringency, followed by Orbitrap MS analysis of mitotic partners. In parallel co-IP experiments, mouse IgG isotype were used as a negative control for non-specific interactors. An extended list of importin beta-1 interactors (272 protein groups) was obtained, which includes known as well as novel importin beta-1 interactors. The full list of all 272 hits is shown in Supplementary Table 1.

Among well-established importin beta-1 interactors we pulled down various classes of factors: i) the GTPase RAN and RANBP1, both  with global roles in mitotic spindle organization and dynamics^9,10,35,36^; ii) importin alpha and importin-7, both of which cooperate with importin beta-1 in nuclear import of particular classes of cargoes in interphase^1–3^; and iii) components of the RRSU complex (RANBP2, SUMO-conjugated RANGAP1, and UBC9/UBE21), as expected from previous studies^14,37^.

The importin beta-1 co-IP also includes proteins with clear mitotic roles. Of note are tubulin beta (Supplementary Table 1), consistent with the evidence that importin beta-1 co-sediments with polymerized MTs in mitotic cells^12^, and NUSAP, a nucleolar protein in interphase that has tubulin-binding and MT-stabilizing activity in mitosis; through these activities NUSAP regulates the formation of asters and long MTs^38,39^. Interestingly, NUSAP is known to interact with both importin beta-1 and importin-7 (consistent with our detection of importin-7 in the importin beta-1 mitotic interactome); these two importins are reported to differentially inhibit NUSAP’s mitotic functions: importin beta-1 blocks aster assembly, while importin-7 enables the formation of small asters but prevents the assembly of long MTs^38^. Also of interest is the presence in the interactome of BUB3, a mitotic spindle checkpoint factor that promotes the stabilization of correct (“end-on”) MT attachments to KTs. BUB3 is required for BUB1 localization to KTs, and the BUB3/BUB1 complex cooperates with other spindle checkpoint factors to prevent premature anaphase onset. Importin-beta-1 was previously found to interact with BUB3, thus preserving it from the ubiquitin/proteasome system and regulating its stability^40^.

Besides these well-characterized factors, we wished to analyse the profile of importin beta-1 mitotic interactors. As a first approach, we examined the list of all 272 identified hits (Supplementary Table 1) by mining the Mitocheck database, built on a genome-wide siRNA-based screening and phenotypic analysis^41^. 195 of our 272 hits were annotated in Mitocheck. Of those, 86 (roughly 44%) were associated with specific mitotic phenotypes (e.g., mitotic arrest or delay; metaphase alignment problems or no metaphase; segregation problems with chromatin bridges or lagging chromosomes; “grape” phenotype, resulting from chromosome segregation defects at mitotic exit) when their coding genes were silenced. Of the remaining annotated genes, 7 (3.6%) gave a predominantly nuclear phenotype (polylobed or otherwise abnormally shaped nuclei, indicating aberrant nuclear reassembly), 10 (5%) affected cytokinesis, and 51 (26.15%) affected more general features (cell death, or, on the contrary, increased cell proliferation, or altered migration properties). With all possible technical reservations inherent to the Mitocheck siRNA-based methodology, these observations support the conclusion that many importin beta-1 interactors identified in our study are indeed required, directly or indirectly, for some aspect of mitosis.

We next inspected the full list of interactors more closely by text-mining in several resources (see Materials and Methods), in order to get more insight into their nature and functions. Table 1 shows a literature-driven selection of importin beta-1 interactors, most of them originally identified as members of other ontogenic pathways, which nevertheless play roles at various steps of mitotic progression and can thus be regarded as moonlighting proteins. A number of these proteins had not been previously associated with importin beta-1.

**Table 1:** Importin beta-1 interacting factors with mitotic functions: literature-driven selection^(a)^.

Importin beta-1 interaction^(b)^					
Group/pathway	Protein ID	Gene name	Protein names	Pubmed	Biogrid
Nuclear import	Q14974	KPNB1	Importin subunit beta-1	Yes	Yes
	P52292	KPNA2	Importin subunit alpha-1	Yes	Yes
	O95373	IPO7	Importin-7	Yes	Yes
RAN GTPase network	P62826	RAN	GTP-binding nuclear protein Ran	Yes	Yes
	P46060	RANGAP1	Ran GTPase-activating protein 1	Yes	Yes
	P43487	RANBP1	Ran-specific GTPase-activating protein 1	Yes	Yes
SUMO modification	P49792	RANBP2	E3 SUMO-protein ligase RanBP2	Yes	Yes
	P63279	UBE2I	SUMO-conjugating enzyme UBC9	Yes	Yes
Mitotic spindle	P07437	TUBB	Tubulin beta chain	Yes	Yes
	Q9BXS6	NUSAP1	Nucleolar spindle-associated protein 1	Yes	Yes
Mitotic checkpoint	O43684	BUB3	Mitotic checkpoint protein BUB3	Yes	Yes
Signal transduction	P62258	YWHAE	14-3-3 protein epsilon	Yes	Yes
Chaperones	P08238	HSP90AB1	Heat shock protein HSP 90-beta	Yes	No
	P14625	HSP90B1	Endoplasmin	Yes	No
Nucleolus	P19338	NCL	Nucleolin	Yes	Yes
Ribosome	P23396	RPS3	40 S ribosomal protein S3	Yes	No
Ribonucleoproteins	P07910	HNRNPC	Heterogeneous nuclear ribonucleoproteins C1/C2	Importin alpha-1	No
	P61978	HNRNPK	Heterogeneous nuclear ribonucleoprotein K	Yes	No
	Q00839	HNRNPU	Heterogeneous nuclear ribonucleoprotein U	Yes	No
RNA binding	Q9UMS4	PRPF19	Pre-mRNA-processing factor 19	No	No
	Q01844	EWSR1	RNA-binding protein EWS	No	No
	Q96PK6	RBM14	RNA-binding protein 14	No	No
	Q07666	KHDRBS1	KH domain-containing RNA-binding, signal transduction-associated protein 1	Yes	No
	P84090	ERH	Enhancer of Rudimentary Homolog	No	No
	Q86V81	ALYREF	THO complex subunit 4	No	No
RNA helicases	P17844	DDX5	Probable ATP-dependent RNA helicase DDDX5	No	No
	Q92841	DDX17	Probable ATP-dependent RNA helicase DDDX17	Yes	No
Elongation factor	P13639	EEF2	Eukaryotic ElongationFactor 2	No	No
Histones	Q92522	H1FX	Histone H1x	No	No
Cytoskeleton	Q15149	PLEC	Plectin	No	No
	P63261	ACTG1	Actin, cytoplasmic 2	No	No
	P60709	ACTB	Actin, cytoplasmic 1	No	No
	P46940	IQGAP1	Ras GTPase-activating-like protein IQGAP1	No	No
Vesicle transport	Q00610	CLTC	Clathrin h)eavy chain 1	Functional	No
	Q92734	TFG	Protein TFG (Trk-fused gene)	No	No
Redox	Q06830	PRDX1	Peroxiredoxin-1	No	No

^(a)^The full list of all 272 hits in the mitotic importin beta-1 co-IP is shown in Supplementary Table 1.

^(b)^Interactions with imporitn beta-1 indicated as PubMed were retrieved from in-depth studies of specific proteins; all proteins reported in the list were also checked in Biogrid (https://thebiogrid.org/).

Some factors in the list have general roles in mitosis. Some of them, with signalling and chaperone activity, were known to bind importin beta-1. The 14–3–3 epsilon protein YWHAE regulates M entry by associating with and regulating the localization of cdc25B^42^, and also cooperates with dynein to maintain spindle orientation^43^. Heat shock proteins have roles in mitotic entry, progression and exit, often - though not exclusively - by regulating the stability of various mitotic proteins^44^. The HSP90B family members identified in our list were reported to interact with, and generally stabilize, G2/M checkpoint factors (e.g., the Chk1, Wee1, and greatwall kinases) and mitotic regulators (Plk1, topoisomearse II-alpha, survivin, and cyclin B1 itself). Some newly identified importin beta-1 interactors also exert global roles in mitosis, including the heterogeneous nuclear ribonucleoprotein C (HNRNPC)^45^, the translation elongation factor EEF2^46^, and protein TFG (Trk-fused gene)^47^: all are involved in regulation of translation, which is essential in mitosis; consistent with this, all - when dysfunctional - yield abnormal mitoses or abrogation of mitosis.

A group of importin beta-1 interactors regulate centrosome function or organization. For example, peroxiredoxin-1 (Prdx1) regulates H2O2 at the centrosome level and is crucial for centrosome function^48^. Prdx1 is inactivated by Cdk1-dependent phosphorylation in early mitosis, thus unshielding mitotic centrosomes from high H_2_O_2_ concentrations, and facilitating the inactivation of centrosomal phosphatases. Dephosphorylation and reactivation of Prdx1 in late mitosis then shields back the centrosome from H_2_O_2_, allowing the local reactivation of phosphatases. The abundance of importin beta-1 at spindle poles^12^, and its ability to interact with Prdx1 detected here, might provide an additional mechanism to regulate the timing of Prdx1 activity/inactivation at centrosomes. Interestingly, the importin beta-1 mitotic interactome also contains catalase (Supplementary Table 1), strengthening the possibility that importin beta-1 might control the redox status during mitosis. RNA-binding protein 14 (RBM14), also acts on centrosome and spindle functions; its inactivation induces ectopic centrosome formation and multipolar spindles^49^, a phenotype also induced by importin beta-1 overepxression. Plectin regulates centrosome positioning in association with BRCA2^50^.

Many identified interactors localize to the spindle, the main site of importin beta-1 localization in mitosis. Clathrin, a component of the endocytic pathway with RNA-binding capacity, and a recognized example of a moonlighting protein, localizes to MTs and regulates spindle functions, in part by localizing other proteins to the spindle^51^. Interestingly, clathrin targets the MT-regulatory factor TACC-3 to the spindle MTs^52^ in an importin-beta-1-sensitive manner: importin beta-1 inhibits clathrin/TACC3 binding, while RANGTP restores it. Despite of this regulatory connection, a direct physical interaction between clathrin and importin beta-1 had not been shown before, suggesting the possibility that importin beta-1 regulates clathrin availability for TACC-3 by binding to it.

In addition, both the ribosomal protein RPS3 and the ribonucleoprotein HNRPU/SAF-A localize at spindles in mitosis and their inactivation yields mitotic delay, spindle defects and chromosome misalignment^53,54^. Interestingly, HNRPU/SAF-A co-immunoprecipitates with Aurora-A and TPX2, which are required for HNRPU/SAF-A association with the spindle; in turn, HNRPU/SAF-A contributes to targeting of Aurora-A to the spindle MTs^55^. Importin beta-1 interacts with and negatively regulates TPX2 activity; the identification of HNRPU/SAF-A in the interactome suggests a possible interplay between importin beta-1, HNRPU/SAF-A, TPX2, and Aurora-A in steps of spindle assembly. Among newly identified importin beta-1 interactors localizing to the spindle, the RNA processing and transport protein EWSR1 is required for mitotic entry and, in cells that do enter mitosis, for proper spindle organization^56,57^. The PRP19 pre-mRNA processing factor affects the mitotic spindle MT density and chromosome alignment^58^. These newly identified interactors might hint at additional mechanisms through which importin beta-1 can regulate the mitotic spindle.

We also identified regulators of MT/KT interactions in the importin beta-1 interactome, the depletion of which generally results in chromosome misalignment and missegregation. A known example is nucleolin, the major nucleolar component, which interacts with the chromosomal periphery in mitosis, including around outer KTs, where it contributes to regulate MT interactions^59^. Noteworthily, nucleolin associates with Hsp90, which contributes to stabilize it^60^, and co-immunoprecipitates with HNRPU/SAF-A^55^: the importin beta-1 interactome contains all these components. Several newly identified importin beta-1 interactors perform similar function. The spliceosome component ERH (Drosophila Enhancer of Rudimentary Homolog), which also interacts with the interactome member RPS3, regulates the levels of the CENP-E kinetochore-associated motor protein^61^, and hence KT interactions with MTs. The THO complex subunit 4 (ALYREF), involved in export of spliced mRNA, is also required for proper chromosome alignment^62^. The RNA helicases (DDX5 and DDX17) regulate the stability of Aurora-B mRNA, and hence the overall Aurora-B protein abundance^63^. The identification of diverse interactors that play roles in chromosome congression and MT/KT interactions thus expands the range of mechanisms that importin beta-1 might regulate.

Finally, the list of importin beta-1 interactors includes factors acting at mitotic exit. Here we identify KHDRBS1/SAM68, known to play roles in mitotic exit^64^. In addition, IQGAP recruits several proteins to the cleavage furrow in cytokinesis^65^;  importantly, it functions in recruitment of NUP98 and other NUPs (MAB414 antibody substrates) during NE reassembly at mitotic exit^66^, a process in which importin beta-1 plays crucial roles^6^. Together, the data identify a variety of factors and pathways potentially regulated by importin beta-1 at various steps during mitosis.

### Automation of a PLA-based method for the validation of interactions

Experimental validation of protein partners after interactomic screening often relies on Western blotting assays of co-immunoprecipitating complexes, or other cell extract-based methods that fail to provide spatial or temporal information on dynamic interactions. We decided to develop an *in situ* proximity ligation assays (is-PLA) protocol to validate true importin beta-1 interactors in mitotic cells and depict where and when the interactions occur. Is-PLA uses ordinary primary antibodies to the proteins of interest, that are revealed by secondary antibodies conjugated to DNA oligonucleotides of complementary sequence: when the target proteins interact, or are in close proximity, the oligonucleotides can be ligated and amplified, and the amplification products are visualized at the site(s) and temporal window in which the interactions occur. We reasoned that the is-PLA method, if developed to be automated, could advance the validation of the interactomic results and add temporal and spatial information on the identified interactions.

**Figure 2:**
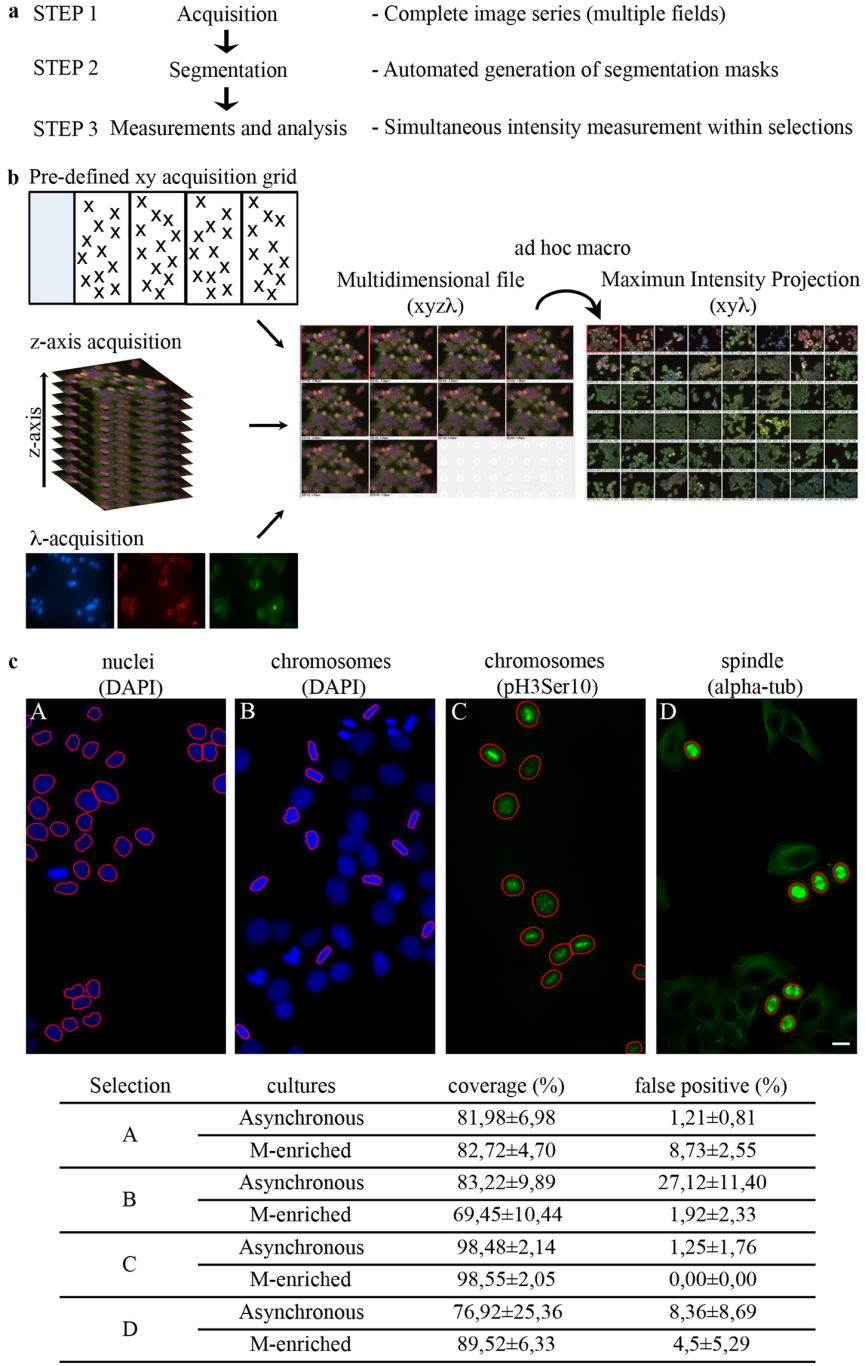
Automated image acquisition. (**a**) The diagram shows the development of a three-step protocol for automated microscopy acquisition and analysis: 1) acquisition of complete image series, giving one multidimensional file as the output; 2) segmentation step, enabling the automated generation of the regions of interest; 3) simultaneous measurements of segmented images in one step. (**b**) Image acquisition protocol. For each PLA combination, a single file is composed of 60 xy points (acquired using a pre-defined xy acquisition grid), each containing the information from 10 z-stacks and 3 wave-lengths (λ: DAPI; FITC; TRITC). The generated multidimensional file (xyzλ) is processed using an ad-hoc macro for the automated generation of the Maximum intensity projection (xyλ), which is then analyzed as described in step 2 and 3. (**c**) Segmentation set-up for automated is-PLA: interphase vs. mitotic figures. Exemplifying segmented images are shown for each selection. The % (± standard deviation) coverage (detected objects over the total number of real objects, determined by manual counting of the same images) and false positives (determined by a manual check of the segmentation process) are indicated in the Table below. At least 3 replicates per condition were analyzed. Bar, 10 μm.

Figure 2a shows a schematics of the is-PLA automation. We combined is-PLA with distinctive labelling markers for mitotic figures, i.e. chromosome labelling (DAPI staining of the DNA, or IF with phospho-Ser10-H3 antibody to the mitotic-specific modification of histone H3), and mitotic spindle visualization (alpha-tubulin IF). Automated image acquisition is schematized in Fig. 2b. Segmentation procedures are used to define single objects within a digital image. We defined intensity, size and circularity thresholds for reproducible image segmentation (see Methods). Panels in Fig. 2c display mitotic cells, automatically distinguished from interphases for their higher DAPI fluorescence intensity and loss of rounded nuclear shape (panel B; compare with interphase nuclei in A), H3 phosphorylation (panel C), and bright stained mitotic spindles (panel D: with the set thresholds for IF intensity and object “roundness”, spindles distinctively stand out from the cytoskeleton of adherent interphase cells). Experiments were set up in both non-synchronized cultures, to control the abundance and localization of the interactions of interest in the absence of checkpoint activation after drug-induced cell cycle arrest, and RO-synchronized cultures to reproduce the conditions used for the interactomic profiling. For all markers, over 70% (non-synchronized cultures) and 80% (mitoses-enriched cultures) of the objects were recognized, with >75% or >90% confidence, respectively (Fig. 2c). Thus, our image acquisition and segmentation workflow yields rapid and accurate detection of mitotic structures in an automated manner.

**Figure 3:**
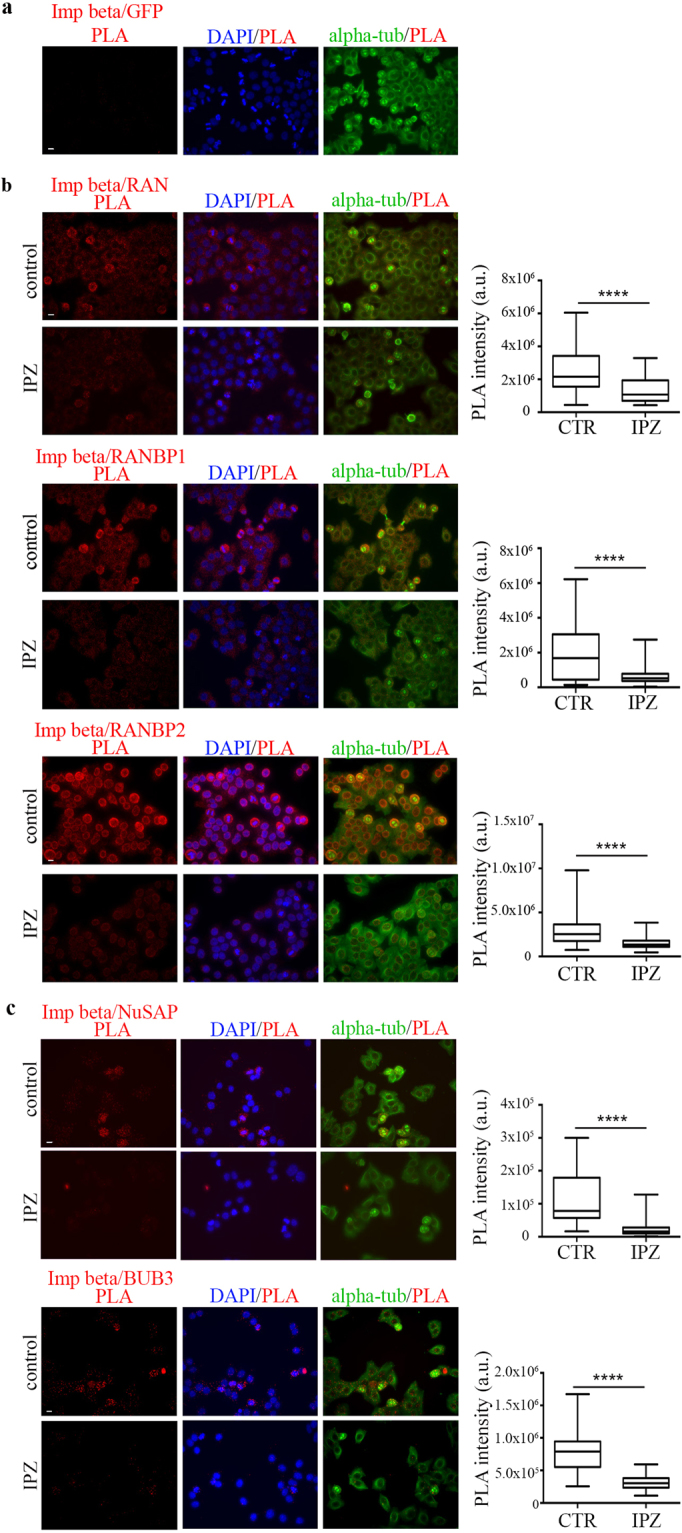
Automated detection of importin beta-1-containing PLA products. (**a)** Negative control for is-PLA experiments: note the absence of PLA signals in non-transfected HeLa cells processed for amplification after incubation with antibodies for importin beta-1 and GFP (not expressed in HeLa cells). (**b)** Fluorescence panels on the left show PLA-positive cells for the interaction between importin beta-1 and well-characterized interactors: RAN, RANBP1 and RANBP2, in untreated cells (control, CTR) and after importazole treatment (IPZ); the box-plot panels on the right represent the distribution of PLA signal intensities (arbitrary units, a.u.) in individual cells. (**c**) PLA-positive cells for the reaction between importin beta-1 and NUSAP or BUB3. Exemplifying panels used for automated measures are shown on the left; on the right, PLA signals are quantified as described for section b. 40 × objective, bar, 10 μm. n counted cells, without and with IPZ were: RAN, 40–35; RANBP1, 190-150; RANBP2, 140-105; NuSAP, 35-30; BUB3, 55-40. ****0.0001, Mann-Whitney test.

Among importin beta-1 interactors to test in PLA assays, we selected RAN, RANBP1 and RANBP2 as established importin beta-1 interactors in all cell cycle stages, and NUSAP and BUB3 as mitotic factors. Two approaches were used to control the specificity of the PLA automated mode. First, as a negative control, native HeLa cells, not expressing GFP, were incubated with importin beta-1 and GFP-specific antibodies, followed by oligonucleotide-conjugated secondary antibodies: no PLA signal was detected, indicating that no aspecific ligation or amplification occurred (Fig. 3a). Second, for each tested reaction, we set up parallel PLAs in cultures treated with importazole, which disrupts importin beta-1 interactions^22^. Results are shown in Fig. 3. Briefly, importin beta-1/RAN interactions (Fig. 3b, upper panel) were detected within the mitotic spindle selection. Mitotic PLA products were similarly detected when testing importin beta-1 in combination with RANBP1 (Fig. 3b, middle panel) and, more abundantly, with RANBP2 (Fig. 3b, lower panel).

The is-PLA protocol also visualized importin-beta-1 interaction products with the spindle-associated protein NUSAP and the spindle checkpoint component BUB3 (Fig. 3c): positive PLA signals were detected for both interactions around the areas of chromosome selection, suggesting an increased abundance in the spindle regions that make contacts with chromosomes. All five PLA reactions were prevented by treatment with importazole. The results demonstrate that the automated PLA mode is reliable and depicts genuine interactions between importin beta-1 and its partners in mitotic cells.

### Localization of importin beta-1 PLA interactions with partners in mitotic cells

During the unfolding of mitosis, importin beta-1 can use different mechanisms to repress its interactors. It inhibits certain MT-regulatory factors, for example TPX2, which it binds via an importin alpha molecule, by co-localizing with them and rendering them inactive in the interaction. In other cases, importin beta-1 displaces factors away from their site of action. For example, it prevents the unscheduled onset of KT functions by localizing certain regulatory factors (e.g., RANBP2) away from KTs^14,29,30^. Depicting the spatial map of importin beta-1 interactions with its partners can help to understand its modes of action in mitotic cells. To fully exploit the informative content of the PLA method, we carried out PLA assays in asynchronous HeLa cultures, where all stages of mitosis are present with physiological frequency, and examined the PLA interaction signals in higher magnification to characterize their localization and abundance with spatiotemporal resolution.

**Figure 4:**
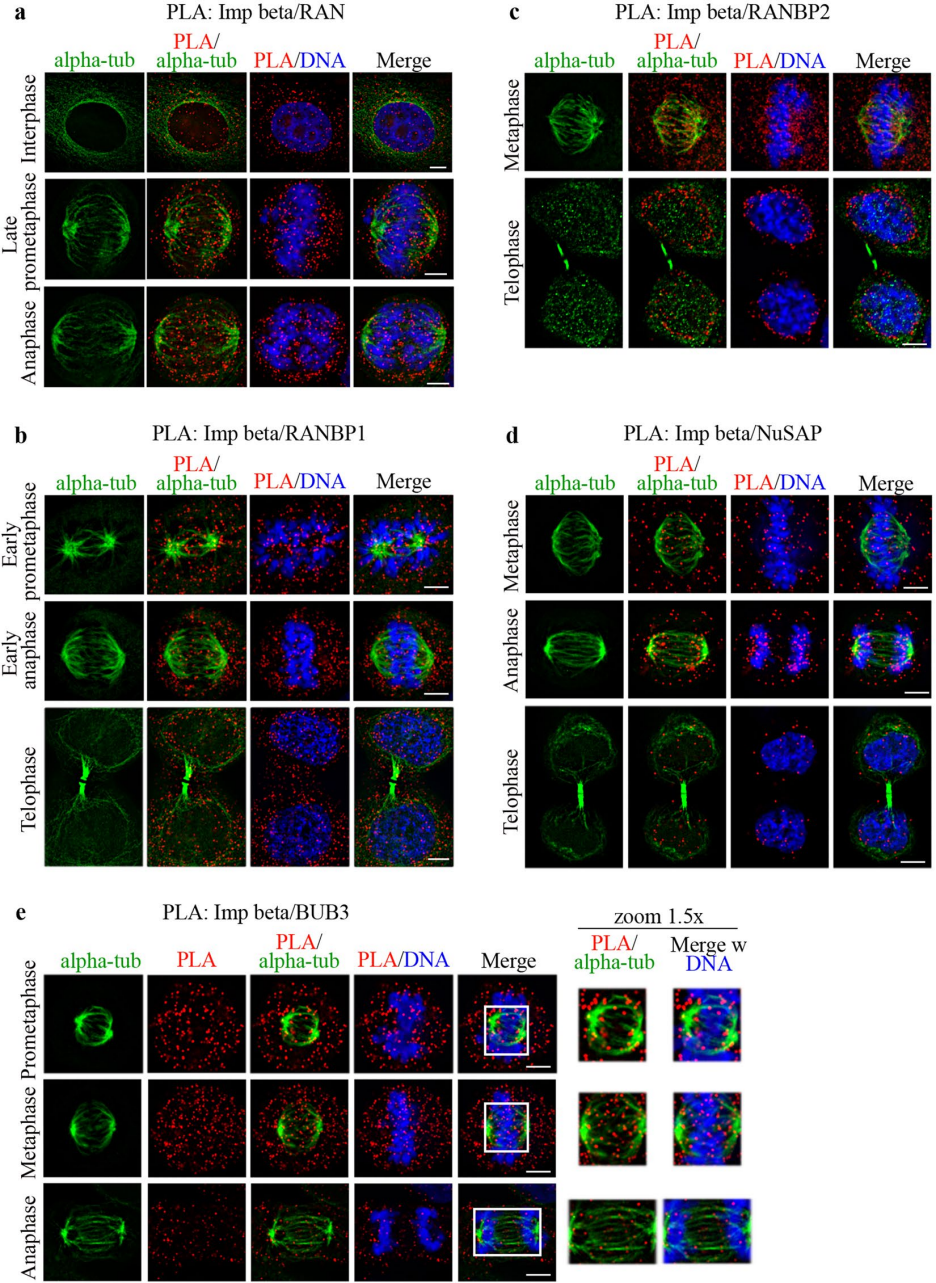
Spatiotemporal resolution of PLA interactions between importin beta-1 and the indicated partners during mitotic progression. PLA panels (100 × objective) depict the sites of interaction of importin beta-1 with partners validated in Fig. 3, relative to the mitotic apparatus (alpha-tubulin), chromosomes or reforming nuclei (DAPI): (**a**) RAN; (**b**) RANBP1; (**c**) RANBP2; (**d**) NUSAP; (**e**) BUB3. Representative stages for each combination are shown and details are given in the text. Bar, 5 μm.

RAN is a highly mobile protein. Previous studies depicted RANGTP enrichment around chromosomes using an indirect method based on FRET reporters for RANGTP-rich territories^13,67^. RANGTP-specific conformational antibody^68^ also detected fractions at centrosomes^69^ and spindle MTs^35^. In PLA assays, RAN/importin beta-1 interactions were visualized around and within the spindle region (Fig. 4a), particularly along MTs (late prometaphase, middle row). PLA signals remained visible at MTs during spindle elongation and chromosome segregation in anaphase (lower row).

We then assessed the mitotic localization of importin beta-1/ RANBP1 PLA products. In early prometaphase, RANBP1/importin beta-1 PLA products were found to distribute throughout the cell volume, yet showed a discrete fraction localizing around asters from which MTs project (Fig. 4b, top row), then persisted in the cytoplasm and at spindle MTs (middle row). In anaphase/early telophase (recognized from the thick midbody), PLA products relocated around the segregating chromosomes (Fig. 4b, bottom row). In more advanced telophase (elongated midbody, decondensed chromatin) PLA products decreased (data not shown), consistent with the notion that RANBP1 is down-regulated at mitotic exit, concomitant with importin beta-1’s resumption of function in nuclear and NE  reorganization^70^.

We next analyzed importin beta-1/RANBP2 PLA products. PLA signals localized along the spindle MTs and at the interface between MT plus-ends and KTs in metaphase (Fig. 4c, top row); chromosomal regions were devoid of PLA signals, consistent with the notion that the KT-associated RANBP2 pool is free of importin beta-1^30^. In telophase, importin beta-1/RANBP2 PLA products relocalized around the decondensing chromatin, in the region where the NE and NPCs reassemble around the reforming nuclei (Fig. 4c, lower row).

PLA analysis of the importin beta-1/NUSAP pair showed a temporally regulated pattern (Fig. 4d). In early mitosis, PLA signals were distributed throughout the cytoplasm, with a small fraction along MTs, suggesting transient dispersal after NE breakdown and nucleolus disassembly (data not shown). The signals then progressively concentrated at MTs until metaphase (Fig. 4d, upper row); most PLA products then remained associated to MTs, while the spindle poles moved apart during anaphase, resulting in the gradual distancing of the PLA product sets during chromosome segregation (middle row). At mitotic exit, PLA signals were visible within nuclei, indicating the resumption of importin beta-1-dependent nuclear import of NUSAP (bottom row). We noticed that intranuclear PLA products have low abundance, suggesting that, once they enter the nucleus, RANGTP readily disassembles them.

Lastly we examined importin beta-1/BUB3 PLA products. As mentioned, importin beta-1 interaction with BUB3 was shown to protect the latter from ubiquitination, and hence proteasome targeting, ultimately modulating the timing of the metaphase-to-anaphase transition^40^. Most studies of BUB3 used GFP-tagged overexpressed chimaeras, and so the endogenous localization is not extensively documented during mitotic progression. We therefore first assessed the precise localization of endogenous, untagged BUB3 in mitotic cells by IF. After mild solubilization, BUB3 was depicted at prometaphase and metaphase KTs (visualized by CREST); its amount increased in cells treated with nocodazole (NOC), which prevented MT assembly and induced sustained activation of the spindle assembly checkpoint (Supplementary Fig. 3). In PLA images (Fig. 4e), many importin beta-1/BUB3 PLA signals are dispersed in the cell volume, with a fraction associated with MTs in prometaphase (Fig. 4e, top row), and in the vicinity of the MT/KT interface in metaphase (middle row). The signals decreased in anaphase (compare the signal abundance during mitotic progression in the PLA column), consistent with the timing at which BUB3 is reported to become accessible to the degradation machinery^39^.

Together, the PLA results for five selected interactions provide an effective validation tool for the co-immunoprecipitating mitotic partners identified in our proteome-wide approach. In addition, they depict spatial and dynamic information for specific pairs of importin beta-1 and its partners, which could not have been obtained otherwise.

## Discussion

Here we report novel information on the versatile ability of importin beta-1 to interact with mitotic partners, which extends and complements studies of interphase transport. We have defined an extensive list of importin beta-1 interacting partners in human mitotic cells. Many proteins identified here are implicated in control of mitosis at various steps and had not been previously associated with importin beta-1. The identification of novel mitotic interactors is relevant, given the implication of importin beta-1 in the generation of mitotic abnormalities in contexts in which it is expressed in a deregulated manner, as in many cancer types^15–20^.

We have identified both known and new targets of importin beta-1. Thirty-five proteins were previously identified as importin beta-1 partners using stable isotope labeling by amino acids in cell cultures (SILAC) in a study of transport pathways^24^, and included essentially ribosomal proteins, nuclear RNPs, and a few proteins acting as nuclear import cargoes in interphase, e.g. nucleolin. Some actually act in mitosis, indicating “moonlighting” roles. We also depicted all components of the RRSU SUMO ligase complex, which is emerging as a temporal regulator of KT functions^28,30,31^, consistent with the established ability of importin beta-1 to interact with RANBP2^14,37^. Another interesting group comprises tubulin beta, the tubulin-binding protein NUSAP and importin-7, with which NUSAP also interacts. These data provide reciprocal evidence to previous studies that identified importin beta-1 in the mitotic MT interactome^71^ and, more recently, in the co-IP of NUSAP, together with the MT-regulatory kinesin MCAK^72^, and demonstrate the reliability of our identification method.

We have searched several resources to obtain specific information on importin beta-1 mitotic interactors. Cross-comparison of our full interactome list (Supplementary Table 1) vs. the Mitocheck database confirms that most importin beta-1 interactors identified here actually play roles in mitosis, as their inactivation is associated with aberrant mitotic phenotypes. Importin beta-1 generally inhibits partners with which it interacts. Our data, therefore, may hint at novel potential pathways of importin beta-1-dependent mitotic control. A number of novel importin beta-1 mitotic interactors (Table 1) include ribonucleoproteins, ribosomal proteins, RNA-binding, splicing and processing factors, and even components of the “translasome” (elongation factors). Mitotic roles are growingly emerging for many proteins of these groups, consistent with the finding that RNAs are part of the mitotic apparatus^73^. Elucidating the precise mechanism by which importin beta-1 acts on these factors, and their regulated pathways, will be important to uncover yet poorly investigated pathways of control of specific mitotic events by importin beta-1.

Cell cycle synchronization protocols were optimised prior to developing our interactomic analysis; nevertheless, due to the highly dynamic nature of mitosis and the rapidity of the prometaphase-metaphase transition, interactions that vary in abundance before or after MT attachment to KTs, such as that involving BUB3 for example, may be over- or under-represented in our list. It was therefore important to validate the interactions identified in the proteomic screening with spatiotemporal resolution in mitotic cells. We report an is-PLA-based automated method that has enabled us to visualize the actual timing and sites at which importin beta-1-containing complexes localize in intact mitotic cells. Our assays show that is-PLA provides a highly informative tool, yielding information that complements that derived from biochemical assays, where detection methods depict the average behaviour of a bulk cell population or cell extract. The integration of these approaches in automated pipelines may represent a key step in high-content screening of protein interactions^74,75^.

High resolution is-PLA assays illustrate the potential of the method to provide visual evidence for functional mechanisms of importin beta-1 with specific partners during mitosis. For example, importin beta-1/NUSAP interactions show variations both in localization and in abundance in mitosis, which may differentially modulate NUSAP activity before, and after, MT attachments to kinetochores. Similarly, we visualized BUB3/importin beta-1 PLA products in early mitosis, which became down-regulated after metaphase, consistent with models for the accessibility of importin beta-1-free BUB3 to the ubiquitination/proteasome machinery past metaphase. More generally, subcellular “territories” in which particular interactions occur can be identified, and predictions for modes of mitotic regulation may be drawn.

In conclusion, the information presented in this work extend our knowledge of importin beta-1 functions available from interphase transport studies and can assist the systematic dissection of the global regulatory roles of importin beta-1 during mitosis.

## Materials and Methods

### Cell cultures

Human HeLa cervix carcinoma epithelial cells were cultured in DMEM supplemented with 10% fetal calf serum, 2% L-glutamine, and 2% penicillin/streptomycin in a humidified atmosphere at 37 °C in 5% CO_2_. Preliminary comparisons of cell cycle synchronization protcols are described in detail in Supplementary Materials and Methods. For proteomic analysis, 5 × 10^6^ HeLa cells were grown in 150 cm^2^ flasks in the presence of RO3306 (9 μM; SML0569, Sigma) for 20 hours, then released in RO3306-free medium, monitored under an inverted microscope and collected at round-up by mechanical shake-off. For automated image acquisition, cells were grown on 4-chamber Culture Slides (Falcon) (8 × 10^4^ cells per 1.7 cm^2^ well), and either left to cycle asynchronously, or synchronized using RO3306 as above and fixed 60 minutes after RO3306 wash-out. Where indicated, cells were treated with 50 μM Importazole (Sigma) for the last 2–3 hours of culture before harvesting the cells.

### Preparation of sepharose-antibody immunoconjugate

To covalently bind antibodies to Protein G-conjugated sepharose beads (Protein G-Sepharose 4 Fast Flow; GE Healthcare), dimethylpimelimidate (DMP; Sigma-Aldrich) was used as crosslinker. Importin beta-1 antibody Sigma I2534 (roughly, 2.5 μg equivalent), or mouse IgG (Santa Cruz Biotechnology sc-2025), were diluted in 1 ml of phosphate buffered saline (PBS), mixed with 15 μl of bead pellet, and incubated overnight at 4 °C on a rotating wheel. The mixture was then centrifuged (3000 rpm, 4 °C, 5 minutes), the pellet was washed with 10 volumes of 0.2 M sodium borate (pH 9.0) and suspended in 100 volumes of 0.2 M sodium borate (pH 9.0)/PBS, to which 20 mM DMP was added just before use. After rotation for 1 hour at room temperature (RT), the beads were washed in 0.2 M ethanolamine (pH 9.0). The remaining reactive amino groups were quenched by adding 0.2 M ethanolamine (pH 9.0) (RT, 2 hours). The bead pellet was then centrifuged (3000 rpm, RT, 5 minutes), washed with 0.1 M glycine (pH 2.5) to remove non-crosslinked antibodies, washed further 3 × in 10 volumes PBS, and stored in PBS at 4 °C.

### Co-immunoprecipitation experiments

The importin beta-1 interactome was captured from mitotic HeLa cells using anti-Importin beta-1 antibody (Sigma-Aldrich I2534). Mouse IgG antibodies (Santa Cruz Biotechnology sc-2025) were used as a control for non-specific binding proteins. RO336-released mitotic cells were centrifuged at low speed, washed twice at 4 °C in cold PBS/0.5 mM PMSF and resuspended in lysis buffer (50 mM Tris HCl (pH 8.0), 150 mM NaCl, 1% NP40) containing protease and phosphatase inhibitors (aprotinin, leupeptin, and pepstatin, all 1 μg/ml; 1 mM PMSF, 50 mM sodium fluoride, 2 mM sodium orthovanadate, 10 mM sodium pyrophosphate). After incubation on ice for 30 minutes, cells were gently homogenized using a Dounce homogenizer and the extent of mechanical disruption of the cells was checked by microscopic observation. After centrifugation (14000 rpm, 4 °C, 20 minutes), the supernatant was quantified by Bradford assay and 1 mg of lysate was immediately precleared with Protein G-Sepharose at 4 °C for 1 hour (15 μl of resin slurry per mg total protein). Importin beta-1 interacting partners were co-immunoprecipitated overnight on a rotating wheel at 4 °C from the precleared mitotic supernatant with anti-importin-beta-1 antibody covalently coupled to Protein G-Sepharose (1 mg protein sample: 2 μg covalent immune-conjugate, home made as described above). For control, the same amount of precleared mitotic extract was incubated with mouse non-specific IgG covalently coupled with Protein G-Sepharose. The mixture was then centrifuged at 3000 rpm, 4 °C for 15 minutes and the pellet was washed 5 × in lysis buffer and again centrifuged as above. After the last centrifugation step, protein complexes were eluted from the beads by a gentle 3 × washing cycle in 15 μl of 0.1 M glycine buffer (pH 2.5) at RT. After centrifugation (3000 rpm, RT, 5 minutes), the pH of the eluted proteins was neutralized by adding 1.5 M Tris HCl pH 8.0.

### Proteomics and data analysis

Aliquots of co-immunoprecipitates were diluted in Laemmli buffer and fractionated through SDS-PAGE on 4–20% Mini-PROTEAN TGX™ gel (Bio-Rad). After colloidal Coomassie staining^76^, gel slices were processed as described^77^ and tryptic peptide mixtures extracted and desalted according to ref.^78^. LC-MS/MS analyses were performed by reverse phase chromatography (C_18_, 5 μm particle size, 200 Å pore size; Magic C18 AQ, Michrom), on an Ultimate3000 system (Dionex, Sunnyvale, CA, USA). Peptides eluted by a two-step gradient of ACN containing 0.1% FA (5–40% in 120 minutes, and 40–85% in 15 minutes) at 300 nl/min flow rate, were directly injected into an LTQ-Orbitrap XL mass spectrometer (Thermo-Fisher Scientific). Data-dependent tandem MS were performed with full precursor ion scans (MS1) collected at 30,000 resolution, with an automatic gain control (AGC) of 1 × 10^6^ ions, and maximal injection time of 1000 ms. The five most intense (>200 counts) ions with charge states ≥ +2 were selected for collision-induced dissociation (CID). Dynamic exclusion was active with 90 ms exclusion for ions selected twice within a 30 ms window. For MS/MS scanning, the minimum MS signal was set to 500, activation time to 30 ms, target value to 10,000 ions, and injection time of 100 ms. All MS/MS spectra were collected using a normalized collision energy of 35% and an isolation window of 2 Th. Spectra were searched against the *Homo sapiens* UniProtKB database (release 2016–07–09, 20154 sequences) and common contaminant proteins using the software package MaxQuant (version 1.5.5.1, Max Planck Institute of Biochemistry, Martinsried, Germany; 64). We set oxidation (methionine) and acetylation (protein N-terminus) as variable modifications, carbamidomethylation (cysteine) as fixed modification, mass tolerance of 20 ppm for the precursor ion (MS) and of 0.5 Da for the fragment ions (MSMS). High-confidence peptide-spectral matches were filtered at <1% false discovery rate. Due to the high sensitivity and resolving power of the mass spectrometric platform used here, together with the high power of the Andromeda search engine in assigning peptide sequences from tandem MS data, the MaxQuant platform yielded a large set of starting data. As a first step of our established proteomic pipeline in data analysis of the original outputs, we discarded identifications for all annotated potential laboratory contaminants (i.e. keratins from experimenters, proteins from reagents etc.), and for hits that the search engine identified in a reverse decoy database used as a false negative control. To extract meaningful information from this cleaned data, a higher confidence matrix was set up by filtering out protein groups recognized with a low confidence level. In particular, we removed proteins which the Andromeda algorithm identified based on an extremely limited number of assigned peptide sequences (i.e.: i) number of unique peptides ≤0, ii) number of peptides ≤1, and iii) only by a peptide carrying a post-translational modification, or iv) through less than 3 MS/MS spectra), or v) presenting an ambiguous association, e.g. associated with multiple genes; see Supplementary Table 1 header legend). Finally, mass spectra intensity difference values between the pulled-down proteins by anti-importin beta-1 and by a non-specific mouse IgG were statistically compared using the Perseus software package (version 1.5.5.3, Max Planck Institute of Biochemistry, Martinsried, Germany^79^) (p-value < 0.05). The resultant list of the 272 high confidence hits was analyzed by data mining against the interphase list^24^, the Mitocheck database version 2.1 (based on EnsEMBL v89, http://www.mitocheck.org/gene.shtml), and the latest releases of UNiProt http://www.uniprot.org/, Biogrid https://thebiogrid.org/, MiCroKITS http://microkit.biocuckoo.org/ and Pubmed https://www.ncbi.nlm.nih.gov/pubmed.

### Immunofluorescence

Cells grown on coverlisps were fixed in 3.7% paraformaldehyde/30 mM sucrose (10 min), permeabilized in 0.1% Triton X-100, then blocked in 3% bovine serum albumin in PBS/0.05% Tween-20 (1 hour, RT). Antibodies used for image segmentation were: chicken anti-alpha-tubulin (Abcam, Ab89984, 1:50), mouse anti-phospho-Ser10-histoneH3 (Millipore, 1:5000). Secondary antibodies were: anti-chicken FITC (Jackson Immunoresearch Laboratories, 1:100), anti-mouse FITC (Jackson Immunoresearch Laboratories, 1:200) and anti-rabbit Cy3 (Jackson Immunoresearch Laboratories, 1:1000). DNA was stained with 0.1 μg/ml 4,6-diamidino-2-phenylindole (DAPI, Sigma Aldrich). Slides were mounted in Vectashield (Vector Laboratories).

### Proximity ligation assay

Cells grown on 4-chamber Culture Slides (Falcon) were processed for PLA in four steps: i) incubation of fixed cell slides with primary specific antibodies for the proteins under examination; ii) incubation with secondary antibodies conjugated with complementary oligonucleotide tails (PLA probes, conventionally called PLUS and MINUS); iii) ligase addition; when the target proteins interact or are very close, the ligation step will produce a DNA circle; iv) rolling circle amplification. All steps were performed using the Duolink^®^ *In Situ* Detection Reagents Red DUO92008 (Sigma-Aldrich). Cells were fixed, blocked, and incubated with primary antibodies as for conventional IF; we used combination of mouse and rabbit primary antibodies for each protein pair (Supplementary Fig. 3). Anti-mouse MINUS and anti-rabbit PLUS PLA probes (PLADuolink^®^ *In Situ* PLA^®^ Probe Anti-Rabbit PLUS, Affinity purified Donkey anti-Rabbit IgG (H + L), DUO92002, and Duolink^®^ *In Situ* PLA^®^ Probe Anti-Mouse MINUS, Affinity purified Donkey anti-Mouse IgG (H + L) DUO92004, respectively, Sigma-Aldrich) were then added (diluted 1:5 in PBS containing 0.05% Tween-20 and 3% bovine serum albumin) and incubated in a pre-heated humidity chamber (60 minutes, 37 °C). Subsequent ligation (30 minutes, 37 °C) and amplification (70 minutes, 37 °C) steps were performed following the Olink Bioscience protocol. To localize PLA signals, cells were co-stained using DAPI and chicken anti-alpha tubulin antibody, followed by FITC-conjugated anti-chicken IgG.

### Image segmentation and measurements

Segmentation is used to partition a digital image to identify objects or extract relevant information. After a background subtraction step, image segmentation on Maximum Intensity Projections was performed using Nis-Elements HC 4.2 (Nikon). We used the Threshold function of the Nis-Elements HC 4.2 software to define regions in the images where we intended to perform the analysis (nuclei, chromosomes, spindles), based on signal intensity, size and circularity. Segmentation masks for chromosomes and spindles were saved and automatically reloaded for each new image set. All steps were performed on a single.nd2 file for the whole experiment acquisition. To evaluate the effectiveness of segmentation, manual analysis of the same images was performed in parallel. For the automated analysis of PLA signals, the “Automated measurements results” function of the software was used: Sum Intensity values for PLA signals within the selected masks were obtained and exported to Excel. Data were statistically analyzed using Graph Pad Prism 6.

### Automated image acquisition

Images from cell samples processed for PLA in 4-chamber culture slides were acquired under a Nikon Eclipse Ti microscope equipped with the Perfect Focus System, a Nikon DS-Qi1 Cooled Digital Monochrome Camera and a 40 × objective (PlanFuor, N.A. 0,75, Nikon), using the Nis-Elements AR 3.2 software. A grid (15 fields per well) was generated, saved and re-loaded for all acquisitions, with a focus adjustment step performed for each new slide. In order to collect the whole spindle-associated signal from rounded-up mitoses, images were acquired over an 8 μm range along the z-dimension (z-step: 0.8 μm). An *ad-hoc* macro was then applied for the automated generation of 2D projection (Maximum Intensity Projection) of all acquired images within a single.nd2 file at the end of the acquisition process.

### Image acquisition for high-resolution PLA analysis

Fixed samples on microscope slides were analyzed under a Nikon Eclipse 90i microscope equipped with a Qicam Fast CCD camera (Qimaging). Single-cell images were taken using an immersion oil 100 × objective (NA 1.3). Images (.nd2 format) were acquired using NIS-Elements AR 3.2 software (Nikon) along the z-axis (0.4 μm stacks, range: 4.4 μm); out of focus planes were uniformly removed before processing. After a background subtraction step, images were deconvolved using the ‘AutoQuant’ deconvolution module of NIS-Element HC (latest release, HC 5.02). Image projections were created using the Extended Depth of Focus (EDF) function of NIS-Element HC 5.02. Focused images were uniformly processed using the LUT (Look Up Table) tool of NIS-Element HC 5.02, and exported in TIFF format for Figure composition using Photoshop CS6.

### Data Availability

The data that support the findings of this study are available from the authors upon reasonable request.

## Electronic supplementary material

Supplementary material
