# Supplementary material for: Visualization of human karyopherin beta-1/importin beta-1 interactions with protein partners in mitotic cells by co-immunoprecipitation and proximity ligation assays
Source: Sci Rep. 2018 Jan 30;8:1850. doi: 10.1038/s41598-018-19351-9 (PMC5789818; doi:10.1038/s41598-018-19351-9)
Supplement: Supplementary file 1 — Supplementary material [file 41598_2018_19351_MOESM1_ESM.pdf]

Visualization of human karyopherin beta-1/importin beta-1 interactions with protein partners in mitotic cells by co-immunoprecipitation and proximity ligation assays

Laura Di Francesco[1,+], Annalisa Verrico[2,+], Italia Anna Asteriti[2], Paola Rovella[2], Pietro Cirigliano[3], Giulia Guarguaglini[2], Maria Eugenia Schininà[1*] and Patrizia Lavia[2*]

[1] Dipartimento di Scienze Biochimiche, Sapienza University of Rome, Piazzale Aldo Moro 5, 00185 Rome, Italy.

[2] Institute of Molecular Biology and Pathology (IBPM), CNR National Research Council of Italy, Via degli Apuli 4, 00185, Rome, Italy

[3] Nikon Instruments S.p.A., Campi Bisenzio, Italy

[+] These authors equally contributed to the work

[*] These authors jointly supervised this work

Contents

Supplementary Table 1

Supplementary Materials and Methods

Supplementary Figures and Legends

Supplementary Table I. Extended list of protein hits identified by nanoLC-MSMS in the Co-IP experiments of importin beta-1 from human mitotic cell extracts (1)

Protein IDs[a]	Majority protein IDs[b]	**Protein names[c]**	Gene names[d]	**Peptides[e]**	Razor + unique peptides[f]	**Unique peptides[g]**	Sequence coverage[h] [%]	Unique + razor sequence coverage[i] [%]	Unique sequence coverage[j] [%]	Mol. weight[k] [kDa]	Score[l]	MS/MS count[m]	**Intensity CoIP anti-ImpB[n]**
Q14974	Q14974	Importin subunit beta-1	KPNB1	**43**	43	**43**	69.4	69.4	69.4	97.169	323.31	679	**3,28E+05**
P63261	P63261	Actin, cytoplasmic 2;Actin, cytoplasmic 2, N-terminally processed	ACTG1	**18**	18	**1**	69.1	69.1	4.5	41.792	323.31	430	**3,14E+06**
P38646	P38646	Stress-70 protein, mitochondrial	HSPA9	**29**	29	**29**	50.8	50.8	50.8	73.68	323.31	270	**3,02E+04**
P35579;A7E2Y1	P35579	Myosin-9	MYH9	**109**	109	**92**	53	53	46.3	226.53	323.31	776	**3,01E+06**
P07910;P0DMR1;O60812;B7ZW38;B2RXH8	P07910	Heterogeneous nuclear ribonucleoproteins C1/C2	HNRNPC	**13**	13	**13**	40.8	40.8	40.8	33.67	181.61	67	**2,90E+06**
Q562R1	Q562R1	Beta-actin-like protein 2	ACTBL2	**3**	1	**1**	11.2	4.8	4.8	42.003	67.061	9	**2,86E+06**
P04083	P04083	Annexin A1	ANXA1	**20**	20	**20**	62.1	62.1	62.1	38.714	323.31	61	**2,86E+06**
Q12905	Q12905	Interleukin enhancer-binding factor 2	ILF2	**19**	19	**19**	69.2	69.2	69.2	43.062	231.62	141	**2,85E+06**
P15924	P15924	Desmoplakin	DSP	**51**	51	**51**	22.1	22.1	22.1	331.77	323.31	523	**2,85E+06**
O43707;Q08043;Q9H254	O43707	Alpha-actinin-4	ACTN4	**42**	42	**25**	58.6	58.6	37.7	104.85	323.31	182	**2,84E+06**
P11532	P11532	Dystrophin	DMD	**3**	3	**3**	1.1	1.1	1.1	426.74	17.253	10	**2,82E+06**
P22626	P22626	Heterogeneous nuclear ribonucleoproteins A2/B1	HNRNPA2B1	**22**	22	**22**	62.6	62.6	62.6	37.429	261.98	125	**2,81E+06**
P14923;P35222	P14923	Junction plakoglobin	JUP	**23**	23	**23**	47.4	47.4	47.4	81.744	323.31	253	**2,78E+06**
P60660;P14649	P60660	Myosin light polypeptide 6	MYL6	**8**	8	**8**	67.5	67.5	67.5	16.93	153.07	45	**2,77E+05**
Q9Y2K3	Q9Y2K3	Myosin-15	MYH15	**2**	2	**2**	1.3	1.3	1.3	224.62	11.534	13	**2,77E+06**
P31943	P31943	Heterogeneous nuclear ribonucleoprotein H;Heterogeneous nuclear ribonucleoprotein H, N-terminally processed	HNRNPH1	**15**	15	**10**	48.1	48.1	31.8	49.229	323.31	89	**2,75E+06**
P06702	P06702	Protein S100-A9	S100A9	**5**	5	**5**	55.3	55.3	55.3	13.242	68.304	67	**2,75E+06**
P46060	P46060	Ran GTPase-activating protein 1	RANGAP1	**23**	23	**23**	44.6	44.6	44.6	63.541	243.93	44	**2,73E+06**

Protein IDs[a]	Majority protein IDs[b]	**Protein names[c]**	Gene names[d]	**Peptides[e]**	Razor + unique peptides[f]	**Unique peptides[g]**	Sequence coverage[h] [%]	Unique + razor sequence coverage[i] [%]	Unique sequence coverage[j] [%]	Mol. weight[k] [kDa]	Score[l]	MS/MS count[m]	**Intensity CoIP anti-ImpB[n]**
P49792;P0DJD0; P0DJD1	P49792	E3 SUMO-protein ligase RanBP2	RANBP2	**46**	46	**37**	20.7	20.7	16.8	358.2	323.31	91	**2,72E+05**
Q02413	Q02413	Desmoglein-1	DSG1	**18**	18	**18**	25.1	25.1	25.1	113.75	255.49	263	**2,71E+06**
P15328	P15328	Folate receptor alpha	FOLR1	**7**	7	**7**	27.2	27.2	27.2	29.819	209.91	48	**2,68E+06**
P62805	P62805	Histone H4	HIST1H4 A	**5**	5	**5**	47.6	47.6	47.6	11.367	46.226	22	**2,66E+06**
P51991	P51991	Heterogeneous nuclear ribonucleoprotein A3	HNRNPA3	**12**	12	**12**	33.1	33.1	33.1	39.594	177.1	44	**2,66E+06**
Q08188	Q08188	Protein-glutamine gamma-glutamyltransferase E;Protein-glutamine gamma-glutamyltransferase E 50 kDa catalytic chain;Protein-glutamine gamma-glutamyltransferase E 27 kDa non-catalytic chain	TGM3	**15**	15	**15**	35.6	35.6	35.6	76.631	323.31	59	**2,64E+06**
P62826	P62826	GTP-binding nuclear protein Ran	RAN	**6**	6	**6**	29.6	29.6	29.6	24.423	48.561	19	**2,64E+06**
P43487	P43487	Ran-specific GTPase-activating protein	RANBP1	**5**	5	**5**	39.3	39.3	39.3	23.31	47.222	13	**2,63E+06**
Q12906;Q96SI9	Q12906	Interleukin enhancer-binding factor 3	ILF3	**20**	20	**20**	34.9	34.9	34.9	95.337	187.81	79	**2,63E+06**
Q14103	Q14103	Heterogeneous nuclear ribonucleoprotein D0	HNRNPD	**7**	6	**6**	20.3	18	18	38.434	83.836	31	**2,63E+06**
P11021	P11021	78 kDa glucose-regulated protein	HSPA5	**24**	24	**23**	41.7	41.7	41.7	72.332	323.31	53	**2,63E+06**
P11940;Q9H361; Q4VXU2;Q5JQF 8;Q96DU9	P11940	Polyadenylate-binding protein 1	PABPC1	**18**	18	**12**	42.1	42.1	27.5	70.67	201.85	40	**2,63E+06**
P60709;A5A3E0	P60709	Actin, cytoplasmic 1;Actin, cytoplasmic 1, N-terminally processed	ACTB	**18**	1	**1**	69.1	4.5	4.5	41.736	86.721	13	**2,63E+06**
Q00839	Q00839	Heterogeneous nuclear ribonucleoprotein U	HNRNPU	**16**	16	**16**	26.5	26.5	26.5	90.583	207.76	86	**2,62E+06**
P51648	P51648	Fatty aldehyde dehydrogenase	ALDH3A2	**13**	13	**12**	27	27	24.5	54.847	154.59	17	**2,62E+05**

Protein IDs[a]	Majority protein IDs[b]	**Protein names[c]**	Gene names[d]	**Peptides[e]**	Razor + unique peptides[f]	**Unique peptides[g]**	Sequence coverage[h] [%]	Unique + razor sequence coverage[i] [%]	Unique sequence coverage[j] [%]	Mol. weight[k] [kDa]	Score[l]	MS/MS count[m]	**Intensity CoIP anti-ImpB[n]**
P11142	P11142	Heat shock cognate 71 kDa protein	HSPA8	**21**	20	**14**	42.9	42.9	32.5	70.897	238.57	60	**2,61E+05**
Q08554	Q08554	Desmocollin-1	DSC1	**11**	11	**11**	16.3	16.3	16.3	99.986	136.03	88	**2,61E+06**
P05109	P05109	Protein S100-A8;Protein S100-A8, N-terminally processed	S100A8	**3**	3	**3**	31.2	31.2	31.2	10.834	61.946	50	**2,60E+06**
P04406	P04406	Glyceraldehyde-3-phosphate dehydrogenase	GAPDH	**11**	11	**11**	46.6	46.6	46.6	36.053	114.64	80	**2,59E+06**
P35637	P35637	RNA-binding protein FUS	FUS	**8**	8	**6**	21.9	21.9	21.9	53.425	55.198	30	**2,59E+06**
Q92734	Q92734	Protein TFG	TFG	**11**	11	**11**	36.5	36.5	36.5	43.447	170.22	43	**2,59E+05**
Q08211	Q08211	ATP-dependent RNA helicase A	DHX9	**37**	37	**37**	38.7	38.7	38.7	140.96	323.31	149	**2,58E+06**
P04792	P04792	Heat shock protein beta-1	HSPB1	**6**	6	**6**	39.5	39.5	39.5	22.782	60.443	10	**2,58E+05**
P04080	P04080	Cystatin-B	CSTB	**3**	3	**3**	45.9	45.9	45.9	11.139	20.553	5	**2,57E+05**
Q06830;Q13162	Q06830	Peroxiredoxin-1	PRDX1	**6**	6	**5**	36.2	36.2	30.7	22.11	37.237	9	**2,56E+06**
P61978	P61978	Heterogeneous nuclear ribonucleoprotein K	HNRNPK	**10**	10	**10**	29.4	29.4	29.4	50.976	201.72	51	**2,56E+06**
Q9NYL9	Q9NYL9	Tropomodulin-3	TMOD3	**13**	13	**13**	48.9	48.9	48.9	39.594	224.27	35	**2,56E+05**
O14979	O14979	Heterogeneous nuclear ribonucleoprotein D-like	HNRNPD L	**9**	9	**8**	19	19	17.1	46.437	130.38	25	**2,55E+06**
Q9UBG3	Q9UBG3	Cornulin	CRNN	**9**	9	**9**	34.1	34.1	34.1	53.533	90.649	15	**2,54E+06**
Q07065	Q07065	Cytoskeleton-associated protein 4	CKAP4	**18**	18	**18**	41.2	41.2	41.2	66.022	228.91	22	**2,54E+06**
P17844	P17844	Probable ATP-dependent RNA helicase DDX5	DDX5	**16**	16	**12**	33.9	33.9	26.2	69.147	154.68	37	**2,54E+06**
Q9UBC9	Q9UBC9	Small proline-rich protein 3	SPRR3	**2**	2	**2**	14.2	14.2	14.2	18.154	15.835	3	**2,53E+06**
Q96DA0	Q96DA0	Zymogen granule protein 16 homolog B	ZG16B	**4**	4	**4**	37	37	37	22.739	122.45	11	**2,51E+06**
Q96P63	Q96P63	Serpin B12	12	**8**	8	**8**	25.4	25.4	25.4	46.276	127.17	72	**2,51E+06**
Q9BUJ2	Q9BUJ2	Heterogeneous nuclear ribonucleoprotein U-like protein 1	HNRNPU L1	**15**	15	**15**	26.2	26.2	26.2	95.737	323.31	45	**2,50E+06**
O43390	O43390	Heterogeneous nuclear ribonucleoprotein R	HNRNPR	**17**	17	**14**	28.9	28.9	24	70.942	128.1	38	**2,50E+06**

Protein IDs[a]	Majority protein IDs[b]	**Protein names[c]**	Gene names[d]	**Peptides[e]**	Razor + unique peptides[f]	**Unique peptides[g]**	Sequence coverage[h] [%]	Unique + razor sequence coverage[i] [%]	Unique sequence coverage[j] [%]	Mol. weight[k] [kDa]	Score[l]	MS/MS count[m]	**Intensity CoIP anti-ImpB[n]**
Q7Z406	Q7Z406	Myosin-14	MYH14	**9**	1	**1**	4.2	0.6	0.6	227.87	7.265	5	**2,50E+06**
P81605	P81605	Dermcidin;Survival-promoting peptide;DCD-1	DCD	**3**	3	**3**	25.5	25.5	25.5	11.284	18.109	13	**2,49E+06**
P06733;P13929;P09104	P06733	Alpha-enolase	ENO1	**12**	12	**12**	41	41	41	47.168	242.85	35	**2,49E+06**
O00159	O00159	Unconventional myosin-Ic	MYO1C	**24**	24	**24**	29.6	29.6	29.6	121.68	304.17	69	**2,49E+06**
P38159;Q96E39; O75526;Q8N7X1	P38159	RNA-binding motif protein, X chromosome;RNA-binding motif protein, X chromosome, N-terminally processed	RBMX	**5**	5	**5**	15.1	15.1	15.1	42.331	54.98	17	**2,48E+05**
P12814;P35609	P12814	Alpha-actinin-1	ACTN1	**37**	20	**20**	56.1	34.6	34.6	103.06	276.79	47	**2,48E+06**
Q99729	Q99729	Heterogeneous nuclear ribonucleoprotein A/B	HNRNPAB	**8**	8	**8**	19.9	19.9	19.9	36.224	84.249	20	**2,48E+06**
P51398	P51398	28S ribosomal protein S29, mitochondrial	DAP3	**12**	12	**12**	41	41	41	45.566	95.312	34	**2,47E+06**
Q96PK6	Q96PK6	RNA-binding protein 14	RBM14	**8**	8	**8**	19.6	19.6	19.6	69.491	74.854	19	**2,47E+06**
Q01469;A8MUU1	Q01469	Fatty acid-binding protein, epidermal	FABP5	**5**	5	**5**	54.1	54.1	54.1	15.164	67.69	13	**2,47E+06**
Q9Y5M8	Q9Y5M8	Signal recognition particle receptor subunit beta	SRPRB	**3**	3	**3**	16.6	16.6	16.6	29.702	21.31	4	**2,47E+06**
O60437	O60437	Periplakin	PPL	**13**	13	**13**	8.8	8.8	8.8	204.74	167.9	13	**2,47E+06**
Q13151	Q13151	Heterogeneous nuclear ribonucleoprotein A0	HNRNPA0	**8**	8	**8**	37	37	37	30.84	133.61	22	**2,46E+06**
Q9NZT1	Q9NZT1	Calmodulin-like protein 5	CALML5	**6**	6	**6**	65.8	65.8	65.8	15.892	93.154	72	**2,46E+06**
P10599	P10599	Thioredoxin	TXN	**2**	2	**2**	23.8	23.8	23.8	11.737	14.926	20	**2,46E+06**
P05089	P05089	Arginase-1	ARG1	**9**	9	**9**	41.9	41.9	41.9	34.735	107.19	82	**2,46E+06**
Q5T750	Q5T750	Skin-specific protein 32	XP32	**2**	2	**2**	8	8	8	26.238	11.928	22	**2,46E+06**
P46782	P46782	40S ribosomal protein S5;40S ribosomal protein S5, N-terminally processed	RPS5	**5**	5	**5**	36.3	36.3	36.3	22.876	88.347	24	**2,46E+06**
P14618	P14618	Pyruvate kinase PKM	PKM	**10**	10	**9**	31.6	31.6	29.6	57.936	156.8	36	**2,46E+06**
P14866	P14866	Heterogeneous nuclear ribonucleoprotein L	HNRNPL	**14**	14	**14**	38.7	38.7	38.7	64.132	159.87	32	**2,46E+06**

Protein IDs[a]	Majority protein IDs[b]	**Protein names[c]**	Gene names[d]	**Peptides[e]**	Razor + unique peptides[f]	**Unique peptides[g]**	Sequence coverage[h] [%]	Unique + razor sequence coverage[i] [%]	Unique sequence coverage[j] [%]	Mol. weight[k] [kDa]	Score[l]	MS/MS count[m]	**Intensity CoIP anti-ImpB[n]**
P25398	P25398	40S ribosomal protein S12	RPS12	**6**	6	**6**	62.1	62.1	62.1	14.515	117.68	34	**2,46E+06**
Q9UKM9	Q9UKM9	RNA-binding protein Raly	RALY	**4**	4	**4**	17.3	17.3	17.3	32.463	46.581	10	**2,45E+06**
P47929	P47929	Galectin-7	LGALS7	**4**	4	**4**	39	39	39	15.075	36.544	17	**2,45E+06**
P12273	P12273	Prolactin-inducible protein	PIP	**5**	5	**5**	36.3	36.3	36.3	16.572	50.43	19	**2,44E+05**
P62269	P62269	40S ribosomal protein S18	RPS18	**5**	5	**5**	30.3	30.3	30.3	17.718	30.772	11	**2,44E+06**
O95373	O95373	Importin-7	IPO7	**15**	15	**15**	18.1	18.1	18.1	119.52	157.22	27	**2,43E+06**
P35268	P35268	60S ribosomal protein L22	RPL22	**3**	3	**3**	30.5	30.5	30.5	14.787	53.805	4	**2,43E+06**
Q13310	Q13310	Polyadenylate-binding protein 4	PABPC4	**12**	6	**6**	25.5	11	11	70.782	35.492	9	**2,42E+06**
Q9Y224	Q9Y224	UPF0568 protein C14orf166	C14orf166	**8**	8	**8**	45.5	45.5	45.5	28.068	142.89	55	**2,42E+06**
Q13867	Q13867	Bleomycin hydrolase	BLMH	**3**	3	**3**	11.2	11.2	11.2	52.562	19.514	18	**2,42E+06**
P35580	P35580	Myosin-10	MYH10	**48**	35	**31**	32.2	25.3	23.3	229	323.31	90	**2,42E+06**
P23396	P23396	40S ribosomal protein S3	RPS3	**11**	11	**11**	57.2	57.2	57.2	26.688	97.429	23	**2,41E+06**
P62158	P62158	Calmodulin	CALM1	**4**	4	**4**	45.6	45.6	45.6	16.837	33.255	9	**2,40E+06**
P08174	P08174	Complement decay-accelerating factor	CD55	**4**	4	**4**	14.7	14.7	14.7	41.4	30.694	11	**2,39E+06**
P31944	P31944	Caspase-14;Caspase-14 subunit p17, mature form;Caspase-14 subunit p10, mature form;Caspase-14 subunit p20, intermediate form;Caspase-14 subunit p8, intermediate form	CASP14	**7**	7	**7**	31.8	31.8	31.8	27.679	106.1	39	**2,39E+06**
P31942	P31942	Heterogeneous nuclear ribonucleoprotein H3	HNRNPH3	**5**	5	**5**	24.9	24.9	24.9	36.926	100.09	20	**2,39E+06**
P63279	P63279	SUMO-conjugating enzyme UBC9	UBE2I	**4**	4	**4**	28.5	28.5	28.5	18.007	24.984	7	**2,38E+06**
P08708	P08708	40S ribosomal protein S17	RPS17	**5**	5	**5**	48.9	48.9	48.9	15.55	83.457	33	**2,38E+06**
P22735	P22735	Protein-glutamine gamma-glutamyltransferase K	TGM1	**5**	5	**5**	8.8	8.8	8.8	89.786	48.331	15	**2,38E+06**
P39019	P39019	40S ribosomal protein S19	RPS19	**5**	5	**5**	29.7	29.7	29.7	16.06	31.777	8	**2,37E+06**
P28799	P28799	Granulins;Acrogranin;Paragranulin;Granulin-1;Granulin-2;Granulin-3;Granulin-4;Granulin-5;Granulin-6;Granulin-7	GRN	**7**	7	**7**	13.5	13.5	13.5	63.544	56.009	17	**2,37E+06**

Protein IDs[a]	Majority protein IDs[b]	**Protein names[c]**	Gene names[d]	**Peptides[e]**	Razor + unique peptides[f]	**Unique peptides[g]**	Sequence coverage[h] [%]	Unique + razor sequence coverage[i] [%]	Unique sequence coverage[j] [%]	Mol. weight[k] [kDa]	Score[l]	MS/MS count[m]	**Intensity CoIP anti-ImpB[n]**
Q92817	Q92817	Envoplakin	EVPL	**11**	11	**11**	7.5	7.5	7.5	231.6	88.591	15	**2,37E+04**
Q13835	Q13835	Plakophilin-1	PKP1	**2**	2	**2**	3.6	3.6	3.6	82.86	19.364	13	**2,36E+06**
P07437;Q9BUF5	P07437	Tubulin beta chain	TUBB	**14**	4	**3**	43.5	13.3	9.9	49.67	97.276	18	**2,36E+06**
P52597	P52597	Heterogeneous nuclear ribonucleoprotein F;Heterogeneous nuclear ribonucleoprotein F, N-terminally processed	HNRNPF	**7**	5	**5**	25.3	18.8	18.8	45.671	98.205	17	**2,36E+06**
P01040	P01040	Cystatin-A;Cystatin-A, N-terminally processed	CSTA	**3**	3	**3**	42.9	42.9	42.9	11.006	22.255	28	**2,36E+06**
P62249	P62249	40S ribosomal protein S16	RPS16	**8**	8	**8**	41.8	41.8	41.8	16.445	46.292	10	**2,36E+06**
Q09666	Q09666	Neuroblast differentiation-associated protein AHNAK	AHNAK	**7**	7	**7**	5.7	5.7	5.7	629.09	43.085	8	**2,35E+06**
P62318	P62318	Small nuclear ribonucleoprotein Sm D3	SNRPD3	**4**	4	**4**	37.3	37.3	37.3	13.916	46.844	7	**2,35E+06**
Q15517	Q15517	Corneodesmosin	CDSN	**2**	2	**2**	7.9	7.9	7.9	51.522	14.035	24	**2,35E+06**
P26599	P26599	Polypyrimidine tract-binding protein 1	PTBP1	**9**	9	**9**	30.1	30.1	30.1	57.221	156.35	45	**2,35E+06**
P08754	P08754	Guanine nucleotide-binding protein G(k) subunit alpha	GNAI3	**8**	8	**3**	32.2	32.2	15.3	40.532	60.575	18	**2,35E+06**
P60866	P60866	40S ribosomal protein S20	RPS20	**3**	3	**3**	22.7	22.7	22.7	13.373	22.866	8	**2,34E+06**
Q969G3	Q969G3	SWI/SNF-related matrix-associated actin-dependent regulator of chromatin subfamily E member 1	SMARCE 1	**7**	7	**7**	24.6	24.6	24.6	46.649	84.073	10	**2,34E+06**
Q14573	Q14573	Inositol 1,4,5-trisphosphate receptor type 3	ITPR3	**2**	2	**2**	1.5	1.5	1.5	304.1	11.601	7	**2,33E+06**
P55084	P55084	Trifunctional enzyme subunit beta, mitochondrial;3-ketoacyl-CoA thiolase	HADHB	**7**	7	**7**	14.6	14.6	14.6	51.294	43.104	7	**2,33E+05**
P55795	P55795	Heterogeneous nuclear ribonucleoprotein H2	HNRNPH 2	**10**	6	**6**	34.1	21.6	21.6	49.263	122.21	21	**2,32E+06**

Protein IDs[a]	Majority protein IDs[b]	**Protein names[c]**	Gene names[d]	**Peptides[e]**	Razor + unique peptides[f]	**Unique peptides[g]**	Sequence coverage[h] [%]	Unique + razor sequence coverage[i] [%]	Unique sequence coverage[j] [%]	Mol. weight[k] [kDa]	Score[l]	MS/MS count[m]	**Intensity CoIP anti-ImpB[n]**
P04040	P04040	Catalase	CAT	**7**	7	**7**	16.1	16.1	16.1	59.755	51.797	32	**2,32E+06**
P08238	P08238	Heat shock protein HSP 90-beta	HSP90AB 1	**7**	7	**3**	11.9	11.9	5.1	83.263	81.294	10	**2,32E+06**
Q1KMD3	Q1KMD3	Heterogeneous nuclear ribonucleoprotein U-like protein 2	HNRNPU L2	**14**	14	**14**	26.1	26.1	26.1	85.104	172.94	37	**2,31E+06**
P63104;P27348;Q 04917;P31946	P63104	14-3-3 protein zeta/delta	YWHAZ	**5**	3	**3**	28.2	20.8	20.8	27.745	179.34	6	**2,31E+06**
P67809;Q9Y2T7	P67809	Nuclease-sensitive element-binding protein 1	YBX1	**5**	5	**4**	28.7	28.7	24.1	35.924	53.734	17	**2,31E+06**
Q92552	Q92552	28S ribosomal protein S27, mitochondrial	MRPS27	**6**	6	**6**	18.1	18.1	18.1	47.611	57.612	9	**2,31E+06**
P62937	P62937	Peptidyl-prolyl cis-trans isomerase A;Peptidyl-prolyl cis-trans isomerase A, N-terminally processed	PPIA	**3**	3	**3**	21.8	21.8	21.8	18.012	21.29	16	**2,31E+06**
P27482	P27482	Calmodulin-like protein 3	CALML3	**4**	4	**4**	35.6	35.6	35.6	16.891	65.389	4	**2,30E+06**
P32969	P32969	60S ribosomal protein L9	RPL9	**6**	6	**6**	43.8	43.8	43.8	21.863	44.929	13	**2,30E+06**
O60506	O60506	Heterogeneous nuclear ribonucleoprotein Q	SYNCRIP	**9**	6	**6**	20.1	15.1	15.1	69.602	126.84	14	**2,30E+06**
P08865	P08865	40S ribosomal protein SA	RPSA	**6**	6	**6**	33.6	33.6	33.6	32.854	139.75	11	**2,30E+06**
P30050	P30050	60S ribosomal protein L12	RPL12	**6**	6	**6**	54.5	54.5	54.5	17.818	58.164	13	**2,30E+06**
Q15717;Q12926; P26378	Q15717	ELAV-like protein 1	ELAVL1	**8**	8	**8**	30.7	30.7	30.7	36.091	73.895	11	**2,30E+06**
P25705	P25705	ATP synthase subunit alpha, mitochondrial	ATP5A1	**9**	9	**9**	23.3	23.3	23.3	59.75	92.195	19	**2,29E+05**
P09211	P09211	Glutathione S-transferase P	GSTP1	**4**	4	**4**	27.1	27.1	27.1	23.356	36.434	6	**2,29E+06**
P23526	P23526	Adenosylhomocysteinase	AHCY	**8**	8	**8**	22.5	22.5	22.5	47.716	46.943	10	**2,29E+05**
P37108	P37108	Signal recognition particle 14 kDa protein	SRP14	**3**	3	**3**	39	39	39	14.57	73.39	20	**2,28E+06**
P84090	P84090	Enhancer of rudimentary homolog	ERH	**2**	2	**2**	26.9	26.9	26.9	12.259	81.007	7	**2,28E+06**
P13639	P13639	Elongation factor 2	EEF2	**4**	4	**4**	8.2	8.2	8.2	95.337	31.272	7	**2,28E+06**
P04843	P04843	Dolichyl-diphosphooligosaccharide--protein glycosyltransferase subunit 1	RPN1	**9**	9	**9**	18.9	18.9	18.9	68.569	68.766	9	**2,28E+06**

Protein IDs[a]	Majority protein IDs[b]	**Protein names[c]**	Gene names[d]	**Peptides[e]**	Razor + unique peptides[f]	**Unique peptides[g]**	Sequence coverage[h] [%]	Unique + razor sequence coverage[i] [%]	Unique sequence coverage[j] [%]	Mol. weight[k] [kDa]	Score[l]	MS/MS count[m]	**Intensity CoIP anti-ImpB[n]**
Q9Y3I0	Q9Y3I0	tRNA-splicing ligase RtcB homolog	RTCB	**9**	9	**9**	23.2	23.2	23.2	55.21	86.878	17	**2,28E+06**
P02814	P02814	Submaxillary gland androgen-regulated protein 3B;Peptide P-A;Peptide D1A	SMR3B	**2**	2	**2**	65.8	65.8	65.8	8.188	14.136	6	**2,28E+06**
Q92841	Q92841	Probable ATP-dependent RNA helicase DDX17	DDX17	**9**	5	**5**	15.8	9.3	9.3	80.272	70.882	11	**2,27E+06**
P62277	P62277	40S ribosomal protein S13	RPS13	**4**	4	**4**	25.2	25.2	25.2	17.222	25.071	7	**2,27E+06**
P08670;P41219;P17661	P08670	Vimentin	VIM	**11**	11	**11**	28.5	28.5	28.5	53.651	76.75	16	**2,27E+06**
Q14444	Q14444	Caprin-1	CAPRIN1	**6**	6	**6**	14.1	14.1	14.1	78.365	58.382	19	**2,27E+06**
P43243	P43243	Matrin-3	MATR3	**10**	10	**10**	19.1	19.1	19.1	94.622	177.29	39	**2,27E+06**
Q08380	Q08380	Galectin-3-binding protein	LGALS3BP	**5**	5	**5**	11.8	11.8	11.8	65.33	76.562	10	**2,26E+06**
Q96FQ6	Q96FQ6	Protein S100-A16	S100A16	**2**	2	**2**	29.1	29.1	29.1	11.801	29.327	3	**2,26E+06**
P02786	P02786	Transferrin receptor protein 1;Transferrin receptor protein 1, serum form	TFRC	**11**	11	**11**	18.4	18.4	18.4	84.87	65.58	11	**2,25E+06**
P62913	P62913	60S ribosomal protein L11	RPL11	**3**	3	**3**	20.8	20.8	20.8	20.252	55.431	5	**2,25E+06**
P23280	P23280	Carbonic anhydrase 6	CA6	**4**	4	**4**	18.8	18.8	18.8	35.366	74.873	7	**2,25E+06**
Q86V81	Q86V81	THO complex subunit 4	ALYREF	**5**	5	**5**	22.2	22.2	22.2	26.888	90.828	11	**2,25E+06**
Q01844	Q01844	RNA-binding protein EWS	EWSR1	**2**	2	**2**	5.8	5.8	5.8	68.477	24.913	11	**2,25E+06**
P18510	P18510	Interleukin-1 receptor antagonist protein	IL1RN	**2**	2	**2**	19.2	19.2	19.2	20.055	128.59	3	**2,24E+05**
P62750	P62750	60S ribosomal protein L23a	RPL23A	**2**	2	**2**	14.7	14.7	14.7	17.695	12.319	4	**2,24E+06**
P08574	P08574	Cytochrome c1, heme protein, mitochondrial	CYC1	**4**	4	**4**	22.5	22.5	22.5	35.422	28.266	9	**2,24E+06**
P09923	P09923	Intestinal-type alkaline phosphatase	ALPI	**10**	6	**6**	26.1	14.8	14.8	56.811	55.643	11	**2,23E+06**
P01037;P01036	P01037	Cystatin-SN	CST1	**3**	3	**3**	32.6	32.6	32.6	16.387	19.555	5	**2,23E+06**
P10809	P10809	60 kDa heat shock protein, mitochondrial	HSPD1	**6**	6	**6**	18.5	18.5	18.5	61.054	54.909	14	**2,22E+06**
P62081	P62081	40S ribosomal protein S7	RPS7	**4**	4	**4**	26.3	26.3	26.3	22.127	34.434	9	**2,22E+06**

Protein IDs[a]	Majority protein IDs[b]	**Protein names[c]**	Gene names[d]	**Peptides[e]**	Razor + unique peptides[f]	**Unique peptides[g]**	Sequence coverage[h] [%]	Unique + razor sequence coverage[i] [%]	Unique sequence coverage[j] [%]	Mol. weight[k] [kDa]	Score[l]	MS/MS count[m]	**Intensity CoIP anti-ImpB[n]**
P16989	P16989	Y-box-binding protein 3	YBX3	**4**	3	**3**	21.2	17.2	17.2	40.089	23.234	5	**2,22E+04**
P62701;P22090;Q8TD47	P62701	40S ribosomal protein S4, X isoform	RPS4X	**9**	9	**9**	36.5	36.5	36.5	29.597	108.04	22	**2,22E+06**
Q9Y6U3	Q9Y6U3	Adseverin	SCIN	**8**	8	**8**	18.2	18.2	18.2	80.488	196.6	17	**2,22E+06**
Q8NBX0	Q8NBX0	Saccharopine dehydrogenase-like oxidoreductase	SCCPDH	**4**	4	**4**	14.7	14.7	14.7	47.151	24.674	5	**2,21E+06**
P25311	P25311	Zinc-alpha-2-glycoprotein	AZGP1	**5**	5	**5**	26.2	26.2	26.2	34.258	32.825	23	**2,21E+06**
P62888	P62888	60S ribosomal protein L30	RPL30	**3**	3	**3**	40.9	40.9	40.9	12.784	21.63	10	**2,21E+06**
P83731	P83731	60S ribosomal protein L24	RPL24	**2**	2	**2**	10.2	10.2	10.2	17.779	13.284	3	**2,21E+06**
P62263	P62263	40S ribosomal protein S14	RPS14	**3**	3	**3**	23.2	23.2	23.2	16.273	19.506	6	**2,21E+06**
Q6UWP8	Q6UWP8	Suprabasin	SBSN	**4**	4	**4**	16.3	16.3	16.3	60.54	27.073	18	**2,20E+06**
P82673	P82673	28S ribosomal protein S35, mitochondrial	MRPS35	**5**	5	**5**	27.2	27.2	27.2	36.844	44.897	12	**2,20E+06**
P48047	P48047	ATP synthase subunit O, mitochondrial	ATP5O	**5**	5	**5**	34.7	34.7	34.7	23.277	41.622	11	**2,20E+06**
P00338	P00338	L-lactate dehydrogenase A chain	LDHA	**2**	2	**2**	9.6	9.6	9.6	36.688	52.022	5	**2,20E+06**
P61160	P61160	Actin-related protein 2	ACTR2	**4**	4	**4**	12.9	12.9	12.9	44.76	31.257	9	**2,20E+06**
P20073	P20073	Annexin A7	ANXA7	**4**	4	**4**	8.8	8.8	8.8	52.739	34.069	9	**2,19E+06**
Q92522	Q92522	Histone H1x	H1FX	**3**	3	**3**	18.8	18.8	18.8	22.487	23.213	5	**2,19E+06**
P52272	P52272	Heterogeneous nuclear ribonucleoprotein M	HNRNPM	**5**	5	**5**	9.7	9.7	9.7	77.515	68.677	9	**2,18E+06**
P46783;Q9NQ39	P46783	40S ribosomal protein S10	RPS10	**3**	3	**3**	23	23	23	18.898	22.8	8	**2,18E+05**
P61247	P61247	40S ribosomal protein S3a	RPS3A	**5**	5	**5**	22	22	22	29.945	82.715	7	**2,17E+06**
P32926	P32926	Desmoglein-3	DSG3	**3**	3	**3**	4.8	4.8	4.8	107.53	22.885	5	**2,17E+06**
P62316	P62316	Small nuclear ribonucleoprotein Sm D2	SNRPD2	**4**	4	**4**	32.2	32.2	32.2	13.527	32.743	12	**2,17E+06**
P62829	P62829	60S ribosomal protein L23	RPL23	**2**	2	**2**	25	25	25	14.865	15.162	5	**2,17E+06**
P23528;Q9Y281	P23528	Cofilin-1	CFL1	**4**	4	**4**	30.7	30.7	30.7	18.502	33.106	7	**2,17E+06**
P06576	P06576	ATP synthase subunit beta, mitochondrial	ATP5B	**4**	4	**4**	11.3	11.3	11.3	56.559	58.815	11	**2,17E+06**
P30740;P50453;O75830	P30740	Leukocyte elastase inhibitor	SERPINB1	**3**	3	**2**	9.5	9.5	6.9	42.741	21.213	4	**2,16E+06**

Protein IDs[a]	Majority protein IDs[b]	**Protein names[c]**	Gene names[d]	**Peptides[e]**	Razor + unique peptides[f]	**Unique peptides[g]**	Sequence coverage[h] [%]	Unique + razor sequence coverage[i] [%]	Unique sequence coverage[j] [%]	Mol. weight[k] [kDa]	Score[l]	MS/MS count[m]	**Intensity CoIP anti-ImpB[n]**
P52209	P52209	6-phosphogluconate dehydrogenase, decarboxylating	PGD	**3**	3	**3**	10.6	10.6	10.6	53.139	30.317	6	**2,16E+06**
P40939	P40939	Trifunctional enzyme subunit alpha, mitochondrial;Long-chain enoyl-CoA hydratase;Long chain 3-hydroxyacyl-CoA dehydrogenase	HADHA	**6**	6	**6**	14.2	14.2	14.2	82.999	61.422	10	**2,16E+06**
P62244	P62244	40S ribosomal protein S15a	RPS15A	**3**	3	**3**	33.1	33.1	33.1	14.839	21.152	6	**2,15E+06**
P26641	P26641	Elongation factor 1-gamma	EEF1G	**2**	2	**2**	6.2	6.2	6.2	50.118	19.447	6	**2,15E+06**
P07476	P07476	Involucrin	IVL	**3**	3	**3**	6.7	6.7	6.7	68.478	21.471	3	**2,15E+06**
P82650	P82650	28S ribosomal protein S22, mitochondrial	MRPS22	**3**	3	**3**	10	10	10	41.28	30.467	6	**2,15E+06**
P82933	P82933	28S ribosomal protein S9, mitochondrial	MRPS9	**4**	4	**4**	12.9	12.9	12.9	45.834	27.249	5	**2,15E+06**
Q14126	Q14126	Desmoglein-2	DSG2	**12**	12	**12**	18.8	18.8	18.8	122.29	94.277	22	**2,14E+06**
P80723	P80723	Brain acid soluble protein 1	BASP1	**3**	3	**3**	35.7	35.7	35.7	22.693	20.331	7	**2,14E+06**
P02810	P02810	Salivary acidic proline-rich phosphoprotein 1/2;Salivary acidic proline-rich phosphoprotein 1/2;Salivary acidic proline-rich phosphoprotein 3/4;Peptide P-C	PRH1	**2**	2	**2**	46.4	46.4	46.4	17.016	16.41	5	**2,14E+06**
P52292	P52292	Importin subunit alpha-1	KPNA2	**4**	4	**4**	12.7	12.7	12.7	57.861	47.68	6	**2,14E+06**
P36952	P36952	Serpin B5	SERPINB5	**2**	2	**2**	7.7	7.7	7.7	42.1	20.074	3	**2,13E+06**
Q15366;Q15365;P57721	Q15366	Poly(rC)-binding protein 2	PCBP2	**3**	3	**3**	13.4	13.4	13.4	38.58	21.799	7	**2,13E+06**
A8K2U0	A8K2U0	Alpha-2-macroglobulin-like protein 1	A2ML1	**3**	3	**3**	2.8	2.8	2.8	161.1	21.216	4	**2,13E+06**
P05386	P05386	60S acidic ribosomal protein P1	RPLP1	**2**	2	**2**	51.8	51.8	51.8	11.514	37.053	12	**2,13E+06**
Q13283	Q13283	Ras GTPase-activating protein-binding protein 1	G3BP1	**6**	6	**5**	18.2	18.2	15.7	52.164	41.42	12	**2,13E+06**
P09012	P09012	U1 small nuclear ribonucleoprotein A	SNRPA	**2**	2	**2**	16.7	16.7	16.7	31.279	14.992	4	**2,12E+06**

Protein IDs[a]	Majority protein IDs[b]	**Protein names[c]**	Gene names[d]	**Peptides[e]**	Razor + unique peptides[f]	**Unique peptides[g]**	Sequence coverage[h] [%]	Unique + razor sequence coverage[i] [%]	Unique sequence coverage[j] [%]	Mol. weight[k] [kDa]	Score[l]	MS/MS count[m]	**Intensity CoIP anti-ImpB[n]**
P62304	P62304	Small nuclear ribonucleoprotein E	SNRPE	**2**	2	**2**	25	25	25	10.803	14.037	4	**2,12E+06**
O15145	O15145	Actin-related protein 2/3 complex subunit 3	ARPC3	**3**	3	**3**	20.8	20.8	20.8	20.546	21.365	5	**2,11E+06**
P62258	P62258	14-3-3 protein epsilon	YWHAE	**7**	7	**5**	33.3	33.3	26.3	29.174	44.114	11	**2,11E+06**
P63244	P63244	Guanine nucleotide-binding protein subunit beta-2-like 1;Guanine nucleotide-binding protein subunit beta-2-like 1, N-terminally processed	GNB2L1	**4**	4	**4**	15.5	15.5	15.5	35.076	23.416	7	**2,11E+06**
Q15149	Q15149	Plectin	PLEC	**19**	19	**19**	5.6	5.6	5.6	531.78	124.16	25	**2,11E+06**
Q96TA1	Q96TA1	Niban-like protein 1	FAM129B	**3**	3	**3**	5	5	5	84.137	31.014	4	**2,11E+06**
Q6PKG0;Q659C4	Q6PKG0	La-related protein 1	LARP1	**8**	8	**8**	12.7	12.7	12.7	123.51	56.847	15	**2,11E+06**
O00425;Q9NZI8; Q9Y6M1	O00425	Insulin-like growth factor 2 mRNA-binding protein 3	IGF2BP3	**5**	5	**5**	11.4	11.4	11.4	63.704	38.647	8	**2,11E+06**
Q9Y3D9	Q9Y3D9	28S ribosomal protein S23, mitochondrial	MRPS23	**2**	2	**2**	11.6	11.6	11.6	21.77	12.179	4	**2,11E+06**
P36542	P36542	ATP synthase subunit gamma, mitochondrial	ATP5C1	**4**	4	**4**	14.4	14.4	14.4	32.996	22.618	4	**2,10E+06**
P62906	P62906	60S ribosomal protein L10a	RPL10A	**4**	4	**4**	22.6	22.6	22.6	24.831	28.041	5	**2,10E+05**
Q00325	Q00325	Phosphate carrier protein, mitochondrial	SLC25A3	**3**	3	**3**	10.2	10.2	10.2	40.094	18.751	3	**2,10E+06**
O14975	O14975	Very long-chain acyl-CoA synthetase	SLC27A2	**3**	3	**3**	5.2	5.2	5.2	70.311	19.944	3	**2,10E+06**
Q96AG4	Q96AG4	Leucine-rich repeat-containing protein 59	LRRC59	**4**	4	**4**	19.5	19.5	19.5	34.93	23.963	6	**2,10E+06**
P21291	P21291	Cysteine and glycine-rich protein 1	CSRP1	**3**	3	**3**	28	28	28	20.567	18.361	5	**2,10E+06**
P07339	P07339	Cathepsin D;Cathepsin D light chain;Cathepsin D heavy chain	CTSD	**5**	5	**5**	18.7	18.7	18.7	44.552	33.492	11	**2,09E+06**
P45880	P45880	Voltage-dependent anion-selective channel protein 2	VDAC2	**4**	4	**4**	21.4	21.4	21.4	31.566	23.455	4	**2,09E+06**

Protein IDs[a]	Majority protein IDs[b]	**Protein names[c]**	Gene names[d]	**Peptides[e]**	Razor + unique peptides[f]	**Unique peptides[g]**	Sequence coverage[h] [%]	Unique + razor sequence coverage[i] [%]	Unique sequence coverage[j] [%]	Mol. weight[k] [kDa]	Score[l]	MS/MS count[m]	**Intensity CoIP anti-ImpB[n]**
Q00610	Q00610	Clathrin heavy chain 1	CLTC	**17**	17	**14**	14.4	14.4	12.5	191.61	136.59	29	**2,09E+06**
Q02878	Q02878	60S ribosomal protein L6	RPL6	**2**	2	**2**	11.1	11.1	11.1	32.728	12.935	8	**2,08E+06**
P08195	P08195	4F2 cell-surface antigen heavy chain	SLC3A2	**2**	2	**2**	3.5	3.5	3.5	67.993	12.308	4	**2,08E+06**
Q96EY7	Q96EY7	Pentatricopeptide repeat domain-containing protein 3, mitochondrial	PTCD3	**2**	2	**2**	4.2	4.2	4.2	78.549	19.746	3	**2,08E+06**
P62241	P62241	40S ribosomal protein S8	RPS8	**2**	2	**2**	13.5	13.5	13.5	24.205	31.562	8	**2,08E+06**
Q8WWM7	Q8WWM7	Ataxin-2-like protein	ATXN2L	**6**	6	**6**	9.1	9.1	9.1	113.37	35.918	10	**2,08E+06**
P27105	P27105	Erythrocyte band 7 integral membrane protein	STOM	**5**	5	**5**	28.1	28.1	28.1	31.73	63.884	11	**2,07E+05**
P29401	P29401	Transketolase	TKT	**3**	3	**3**	7.9	7.9	7.9	67.877	19.136	4	**2,07E+06**
Q8NCA5	Q8NCA5	Protein FAM98A	FAM98A	**3**	3	**3**	9.8	9.8	9.8	55.4	19.399	6	**2,07E+06**
Q9BXS6	Q9BXS6	Nucleolar and spindle-associated protein 1	NUSAP1	**4**	4	**4**	12.2	12.2	12.2	49.451	22.489	4	**2,07E+06**
Q5BKZ1	Q5BKZ1	DBIRD complex subunit ZNF326	ZNF326	**2**	2	**2**	4.8	4.8	4.8	65.653	12.134	4	**2,06E+06**
Q92922	Q92922	SWI/SNF complex subunit SMARCC1	SMARCC 1	**9**	9	**6**	12.5	12.5	9.4	122.87	66.84	15	**2,06E+06**
Q13405	Q13405	39S ribosomal protein L49, mitochondrial	MRPL49	**2**	2	**2**	16.3	16.3	16.3	19.198	15.528	3	**2,06E+06**
P32119	P32119	Peroxiredoxin-2	PRDX2	**2**	1	**1**	14.1	8.6	8.6	21.892	8.182	5	**2,05E+06**
P62873;Q9HAV0	P62873	Guanine nucleotide-binding protein G(I)/G(S)/G(T) subunit beta-1	GNB1	**3**	3	**2**	11.2	11.2	7.9	37.377	20.72	4	**2,05E+06**
O43175	O43175	D-3-phosphoglycerate dehydrogenase	PHGDH	**4**	4	**4**	10.3	10.3	10.3	56.65	24.427	5	**2,05E+06**
Q96C19;Q9BUP0	Q96C19	EF-hand domain-containing protein D2	EFHD2	**5**	5	**5**	23.3	23.3	23.3	26.697	57.639	6	**2,05E+06**
Q15233	Q15233	Non-POU domain-containing octamer-binding protein	NONO	**3**	3	**3**	7.2	7.2	7.2	54.231	17.531	5	**2,05E+06**
O43795;Q9UBC5	O43795	Unconventional myosin-Ib	MYO1B	**10**	10	**10**	10.5	10.5	10.5	131.98	87.773	14	**2,04E+06**

Protein IDs[a]	Majority protein IDs[b]	**Protein names[c]**	Gene names[d]	**Peptides[e]**	Razor + unique peptides[f]	**Unique peptides[g]**	Sequence coverage[h] [%]	Unique + razor sequence coverage[i] [%]	Unique sequence coverage[j] [%]	Mol. weight[k] [kDa]	Score[l]	MS/MS count[m]	**Intensity CoIP anti-ImpB[n]**
P52815	P52815	39S ribosomal protein L12, mitochondrial	MRPL12	**3**	3	**3**	28.8	28.8	28.8	21.348	20.943	6	**2,04E+06**
Q9UN86	Q9UN86	Ras GTPase-activating protein-binding protein 2	G3BP2	**3**	2	**2**	11.8	9.3	9.3	54.12	13.377	4	**2,04E+06**
P10620	P10620	Microsomal glutathione S-transferase 1	MGST1	**3**	3	**3**	19.4	19.4	19.4	17.598	18.359	4	**2,04E+06**
P14625	P14625	Endoplasmin	HSP90B1	**6**	5	**5**	11.3	9.8	9.8	92.468	77.149	8	**2,04E+06**
P19338	P19338	Nucleolin	NCL	**8**	8	**8**	15.4	15.4	15.4	76.613	63.052	20	**2,03E+06**
P26196	P26196	Probable ATP-dependent RNA helicase DDX6	DDX6	**3**	3	**3**	11.4	11.4	11.4	54.416	19.525	3	**2,02E+06**
P09661	P09661	U2 small nuclear ribonucleoprotein A	SNRPA1	**2**	2	**2**	12.2	12.2	12.2	28.415	15.18	6	**2,02E+06**
P18124	P18124	60S ribosomal protein L7	RPL7	**2**	2	**2**	12.1	12.1	12.1	29.225	12.773	7	**2,02E+06**
P63220	P63220	40S ribosomal protein S21	RPS21	**2**	2	**2**	28.9	28.9	28.9	9.111	12.158	3	**2,02E+06**
Q9NZB2	Q9NZB2	Constitutive coactivator of PPAR-gamma-like protein 1	FAM120A	**4**	4	**4**	4.3	4.3	4.3	121.89	26.294	9	**2,01E+06**
O43148	O43148	mRNA cap guanine-N7 methyltransferase	RNMT	**5**	5	**5**	13.9	13.9	13.9	54.843	41.719	4	**2,01E+06**
P15880	P15880	40S ribosomal protein S2	RPS2	**2**	2	**2**	8.2	8.2	8.2	31.324	12.977	8	**2,01E+06**
P38919	P38919	Eukaryotic initiation factor 4A-III;Eukaryotic initiation factor 4A-III, N-terminally processed	EIF4A3	**2**	2	**2**	3.6	3.6	3.6	46.871	11.843	3	**2,01E+06**
Q9Y399	Q9Y399	28S ribosomal protein S2, mitochondrial	MRPS2	**3**	3	**3**	8.1	8.1	8.1	33.249	16.562	3	**2,00E+06**
P34897	P34897	Serine hydroxymethyltransferase, mitochondrial	SHMT2	**2**	2	**2**	5	5	5	55.992	11.478	4	**2,00E+06**
P46940	P46940	Ras GTPase-activating-like protein IQGAP1	IQGAP1	**5**	5	**5**	4.5	4.5	4.5	189.25	31.111	7	**2,00E+06**
P59998	P59998	Actin-related protein 2/3 complex subunit 4	ARPC4	**4**	4	**4**	29.8	29.8	29.8	19.667	45.345	11	**1,99E+06**
Q9P035	Q9P035	Very-long-chain (3R)-3-hydroxyacyl-CoA dehydratase 3	HACD3	**3**	3	**3**	12.4	12.4	12.4	43.159	17.553	4	**1,99E+06**

Protein IDs[a]	Majority protein IDs[b]	**Protein names[c]**	Gene names[d]	**Peptides[e]**	Razor + unique peptides[f]	**Unique peptides[g]**	Sequence coverage[h] [%]	Unique + razor sequence coverage[i] [%]	Unique sequence coverage[j] [%]	Mol. weight[k] [kDa]	Score[l]	MS/MS count[m]	**Intensity CoIP anti-ImpB[n]**
Q92900	Q92900	Regulator of nonsense transcripts 1	UPF1	**5**	5	**5**	7	7	7	124.34	30.075	8	**1,99E+06**
Q96E29	Q96E29	Transcription termination factor 3, mitochondrial	MTERF3	**3**	3	**3**	9.6	9.6	9.6	47.971	18.681	3	**1,98E+06**
Q8TAQ2	Q8TAQ2	SWI/SNF complex subunit SMARCC2	SMARCC2	**6**	3	**3**	8.4	5.6	5.6	132.88	33.825	13	**1,97E+06**
Q92499	Q92499	ATP-dependent RNA helicase DDX1	DDX1	**8**	8	**8**	13.9	13.9	13.9	82.431	62.164	9	**1,97E+06**
P62280	P62280	40S ribosomal protein S11	RPS11	**3**	3	**3**	22.8	22.8	22.8	18.431	17.717	4	**1,97E+06**
Q6YHK3	Q6YHK3	CD109 antigen	CD109	**4**	4	**4**	4.6	4.6	4.6	161.69	27.318	5	**1,96E+06**
Q9Y2R5	Q9Y2R5	28S ribosomal protein S17, mitochondrial	MRPS17	**2**	2	**2**	29.2	29.2	29.2	14.502	14.309	4	**1,96E+05**
Q02543	Q02543	60S ribosomal protein L18a	RPL18A	**2**	2	**2**	15.3	15.3	15.3	20.762	14.235	3	**1,96E+05**
Q8NC51	Q8NC51	Plasminogen activator inhibitor 1 RNA-binding protein	SERBP1	**2**	2	**2**	8.3	8.3	8.3	44.965	53.191	5	**1,96E+06**
Q9UMS4	Q9UMS4	Pre-mRNA-processing factor 19	PRPF19	**3**	3	**3**	7.9	7.9	7.9	55.18	17.883	5	**1,95E+06**
Q07666	Q07666	KH domain-containing, RNA-binding, signal transduction-associated protein 1	KHDRBS1	**2**	2	**2**	12.2	12.2	12.2	48.227	12.152	3	**1,95E+06**
O43684	O43684	Mitotic checkpoint protein BUB3	BUB3	**2**	2	**2**	8.2	8.2	8.2	37.154	12.896	3	**1,94E+06**
P35232	P35232	Prohibitin	PHB	**3**	3	**3**	18	18	18	29.804	20.183	4	**1,92E+06**
P06753	P06753	Tropomyosin alpha-3 chain	TPM3	**4**	2	**2**	7.7	3.9	3.9	32.95	12.314	3	**1,92E+06**
P05455	P05455	Lupus La protein	SSB	**3**	3	**3**	10	10	10	46.836	26.258	7	**1,91E+06**
P47756	P47756	F-actin-capping protein subunit beta	CAPZB	**3**	3	**3**	13.4	13.4	13.4	31.35	22.582	4	**1,91E+06**
P31327	P31327	Carbamoyl-phosphate synthase [ammonia], mitochondrial	CPS1	**10**	10	**10**	9	9	9	164.94	59.867	12	**1,90E+06**
P27635;Q96L21	P27635	60S ribosomal protein L10	RPL10	**3**	3	**3**	22.4	22.4	22.4	24.604	19.362	4	**1,87E+06**
Q9H2U1;Q6P158	Q9H2U1	ATP-dependent RNA helicase DHX36	DHX36	**5**	5	**5**	5.5	5.5	5.5	114.76	30.839	8	**1,86E+05**
O43143	O43143	Pre-mRNA-splicing factor ATP-dependent RNA helicase DHX15	DHX15	**2**	2	**2**	3.1	3.1	3.1	90.932	20.258	4	**1,85E+06**

Protein IDs[a]	Majority protein IDs[b]	**Protein names[c]**	Gene names[d]	**Peptides[e]**	Razor + unique peptides[f]	**Unique peptides[g]**	Sequence coverage[h] [%]	Unique + razor sequence coverage[i] [%]	Unique sequence coverage[j] [%]	Mol. weight[k] [kDa]	Score[l]	MS/MS count[m]	**Intensity CoIP anti-ImpB[n]**
P53992	P53992	Protein transport protein Sec24C	SEC24C	**8**	8	**8**	11.3	11.3	11.3	118.32	56.353	14	**1,84E+06**
P30040	P30040	Endoplasmic reticulum resident protein 29	ERP29	**2**	2	**2**	9.6	9.6	9.6	28.993	13.226	5	**1,81E+06**
P61981	P61981	14-3-3 protein gamma;14-3-3 protein gamma, N-terminally processed	YWHAG	**3**	1	**1**	13	5.7	5.7	28.302	10.268	3	**1,78E+06**
Q96EP5	Q96EP5	DAZ-associated protein 1	DAZAP1	**2**	2	**2**	7.6	7.6	7.6	43.383	40.612	6	**1,78E+06**
Q15436;Q15437	Q15436	Protein transport protein Sec23A	SEC23A	**7**	7	**7**	12.5	12.5	12.5	86.16	71.149	10	**1,77E+06**

(1) Herein we followed the definitions and parameters given in MaxQuant (ver. 1.5.5.1) for all columns. Columns displaying key information for protein identification are in bold.

[a] Identifier(s) of protein(s) contained in the protein group identified by the MaxQuant algorithm.

[b] IDs of major protein assigned to a protein group.

[c] Name(s) of protein(s) contained within the group.

[d] Name(s) of the gene(s) associated with the protein(s) contained within the protein group. In this study, we only listed protein identifications univocally associated with one gene only.

[e] The total number of peptide sequences associated with all proteins in the group. With the sequence coverage, can be used to qualitatively estimate the level of confidence in protein(s) identification(s). In this study, we accepted from the original data set only identifications achieved with at least 2 peptides.

[f] Sum of the number of peptide sequences exclusively assigned to the protein group (unique peptides), and that of the peptide sequences shared with other protein groups ("razor peptides"). This parameter is used to determine the intensity values.

[g] The total number of peptide sequences exclusively assigned to the protein group. With the sequence coverage, can be used to qualitatively estimate the level of confidence in protein(s) identification(s). In this study, we accepted from the original data set only identification achieved with at least 1 unique peptide.

[h] Sequence coverage of the best protein sequence contained in the group by the identified peptides, expressed as %.

[i] Sequence coverage of the best protein sequence contained in the group by the identified unique and razor peptides, expressed as %.

[j] Sequence coverage of the best protein sequence contained in the group by the identified unique peptides, expressed as %.

[k] Molecular weight of the leading protein sequence contained in the protein group.

[l] Score for the protein identification, determined by multiplying each peptide posterior error probabilities (PEPs) calculated by the Andromeda search engine for assigning statistical significance to the peptide identifications. The larger this score, the more certain is the identification of a protein. The data processing applied in this work (see Material and Methods) goes with proteins identifications with score values >5.

[m] Number of MS/MS spectra used to identified the peptide sequences assigned to the protein group. Used to qualitatively estimate the level of confidence in peptide(s) identification(s). In this study, the original data set has been filtered for identification achieved with at least 3 MS/MS counts.

[n] Summed up eXtracted Ion Current (XIC) of all isotopic clusters associated with the identified peptides recovered ('extracted') from the entire data set for the chromatographic runs. Due to the broad difference in ion current efficiency among peptide sequences, this parameter does not indicate the absolute abunparameteredance of the protein group in a proteomic mixture, but can be used to compare relative abundances, i.e. the ratio of intensities of the assigned peptides across samples (conditions) that are being investigated. Here, the conditions under comparison are anti-imp beta antibody co-IP vs. non specific antibody co-IP, and values are expressed as log2.

Supplementary Materials and Methods

Selection of antibodies for importin beta-1 co-immunoprecipitation

Antibodies raised against various human importin beta-1 epitopes were tested: 1, Abcam 45938 (rabbit polyclonal, fragment 50-150); 2, BD clone 23 (mouse, fragment 48-241); 3, Abcam 45901 (rabbit polyclonal, fragment 500-600); 4, Abcam 2811 (mouse monoclonal, full-length protein); 5, Sigma I2534 (mouse monoclonal, full-length protein). Antibodies were incubated with 1 mg of whole cell extracts (WCE) prepared from mitotic cells (thymidine-synchronized and collected by mitotic shake-off). The loaded WCE sample (40 μg) represents 4% of the WCE input. The antibody pull-down efficiency was evaluated from the intensity ratio of the E1 elution band relative to that of [E1+ SUP] bands. To compare qualitatively the co-IP of antibodies 4, 5, and 2, mitotic WCE (1 mg) were incubated with bead-bound or -unbound antibodies. The recovered co-IP after low stringency salt wash (150 mM NaCl) was fractionated through 10% SDS-polyacrylamide gel electrophoresis. After staining with Blue Silver, prominent bands were excised and processed for reduction, alkylation, trypsin digestion and Maldi-TOF analysis.

Cell cycle synchronization

Protocols to obtain mitosis-enriched cultures were compared after cell cycle arrest and release from: i) 2 mM thymidine (T1895, Sigma) followed by release in medium containing 30 μM deoxycytidine (D3897, Sigma) for 10 hours; ii) the monastrol derivative Eg5 inhibitor STLC (S-trityl-L-cysteine, 2 μM) (164739, Sigma) and release in fresh STLC-free medium for 30-60 minutes; or iii) the ATP-competitive inhibitor of CDK1, RO3306 (9 μM, 20 hours) (SML0569, Sigma) and release in fresh RO3306-free medium for 30-60 minutes. In most experiments in this study, the RO3306 synchronization/release protocol was used. For WB analysis mitotic cells were collected by mechanical shake-off.

BUB3 immunolocalization. Cells were pre-treated with MT-stabilizing buffer, MTSB (4M glycerol, 100 mM Pipes, 1 mM EGTA, 5 mM $MgCl_2$), followed by 0.05% Triton X-100 in MTSB, then fixed in 3.7% para-formaldehyde and permeabilized in ice-cold Methanol. BUB3 was stained with rabbit antibody (Abcam ab133699, 1:250); kinetochores (KTs) were detected using human CREST serum (Antibodies Inc. 15-234-0001, 1:20); microtubules (MTs) were detected using mouse alpha-tubulin antibody (Sigma T5168, 1:3000 dilution). Secondary antibodies were: Cy3-conjugated anti-rabbit IgG for BUB3 (red); 7-amino-4-methylcoumarin-3-acetic acid (AMCA)-

conjugated anti-human IgG for CREST (blue), and fluorescein isothiocyanate (FITC)-conjugated anti-mouse IgG for alpha-tubulin (green) (all from Jackson Immunoresearch Laboratories). Coverslips were mounted in Vectashield (Vector Laboratories).

Western blotting. Protein extracts were separated through SDS-PAGE, transferred to nitrocellulose filters, blocked and incubated with primary antibodies indicated in Supplementary Figure 3. Horseradish peroxidase-conjugated secondary antibodies (goat anti-mouse 1:10.000, goat anti-rabbit 1:10.000 from Santa Cruz Biotechology) were revealed with enhanced chemiluminescence (ECL).

Supplementary Figure legends

Supplementary Figure 1. Selection of importin beta-1 antibodies to profile the importin beta-1 mitotic importome. **a,** Schematic representation of protein-binding domains (double arrowed regions) in the importin beta-1 linear map**.** The lines below identify importin beta-1 fragments used as immunogens for the antibodies under comparison (listed in Supplementary Materials and Methods). **b,** Importin beta-1 yield in IP using the antibodies shown in **a** (cross-linked to the beads) from the whole cell extract input (WCE, 1 mg) prepared from mitotic cells collected by shake-off after thymidine arrest and release. For each antibody the following samples were loaded alongside: WCE, 40 μg; Sup, supernatant (1/20 of the total volume); E1, elution 1 (entire yield). After PAGE fractionation and transfer, all filters were incubated with the indicated antibodies in routine Western immunoblotting. **c,** Low scale MALDI TOF analysis of importin beta-1 co-IP from thymidine arrested and released cells using three antibodies selected in **b** after electrophoresis and excision of most prominent bands; green stars, identified proteins; red arrowheads, unidentified bands.

Supplementary Figure 2. Comparison of cell cycle synchronization methods for preparation of mitotic cell extracts. Cell cycle arrest and release protocols are schematized at the top of each panel; in all cases. For Western blot analysis, mitotic cells were collected after various periods of release from synchronization by mechanical "shake-off" as they detached from the culture flasks at round-up. Harvested cell samples after release were analyzed by Western blot for cyclin B1 expression and by immunofluorescence (IF) staining to examine the mitotic apparatus (alpha-tubulin green) and chromosomes (DAPI, blue) under a 40 x objective. **a,** In thymidine-arrested and released cells, most mitotic figures are normal (IF panels) but mitotic progression is heterogeneous:

histograms in the graph represent the frequency of mitotic substages in samples collected 10 hours after release from thymidine arrest. P+PM, prophase and prometaphase; M, metaphase; A+T, anaphase and telophase. In **b** and **c**, RO3306- and STLC-arrested and released cultures showed higher synchrony in progression through mitosis compared to thymidine and were examined for the presence of abnormal figures by IF. In **c**, STLC arrests the cell cycle relatively later compared with RO3306 (many cells are beyond NEB in STLC-arrested cultures); the graph represents the frequency of abnormal figures among all mitoses and shows that mitoses with monopolar spindles still represent 37,33% of all cells 60 minutes after STLC release. In **b,** most mitotic figures showed a normal mitotic apparatus 60 minutes after release from RO3306 arrest, which was therefore used as the preferred protocol. Bar, 10 µm.

Supplementary Figure 3. The table at the top lists details and specificities of primary antibodies selected for PLA assays. Below, the specificity of the indicated antibodies was tested by Western immunoblotting (40 µg of HeLa mitotic cell extract per lane).

Supplementary Figure 4. Immunolocalization of BUB3 in stages of mitotic progression in physiological mitosis from asynchronously cycling cell cultures (100 x objective)**. a,** abundant BUB3 signals (red) co-localize with KTs, visualized by CREST antibody (blue): **b,** BUB3 signals are gradually lost from KTs as proper attachments with MTs (green) are established. **c,** note the decreased abundance and delocalization of BUB3 signals from KTs in anaphase. **d**, MTs were inhibited by pretreatment with nocodazole (NOC), thus leaving KTs free of attachments: BUB3 signals concentrated at unattached KTs and displayed increased abundance. Bar, 5 µm.

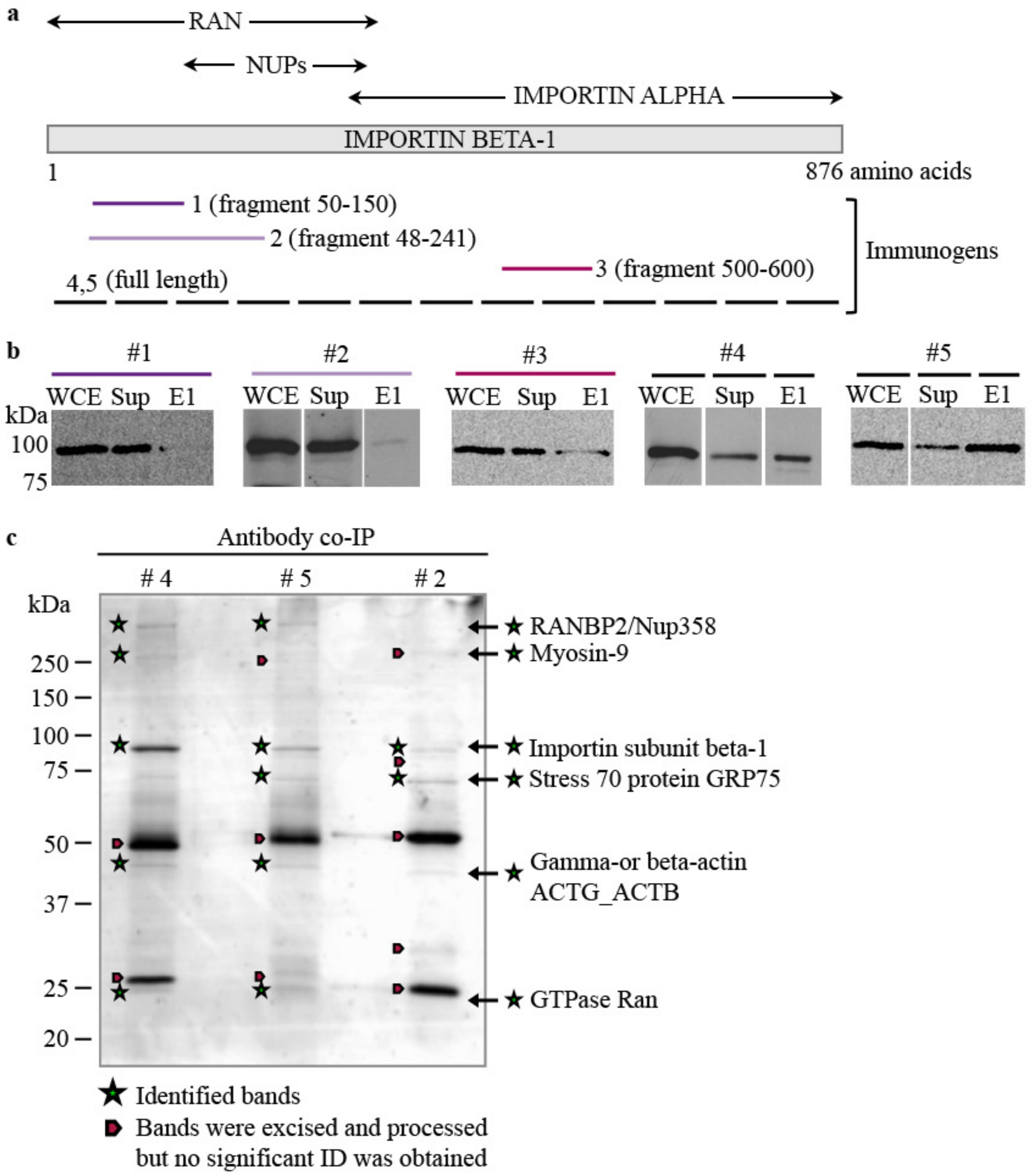

Supplementary figure 1

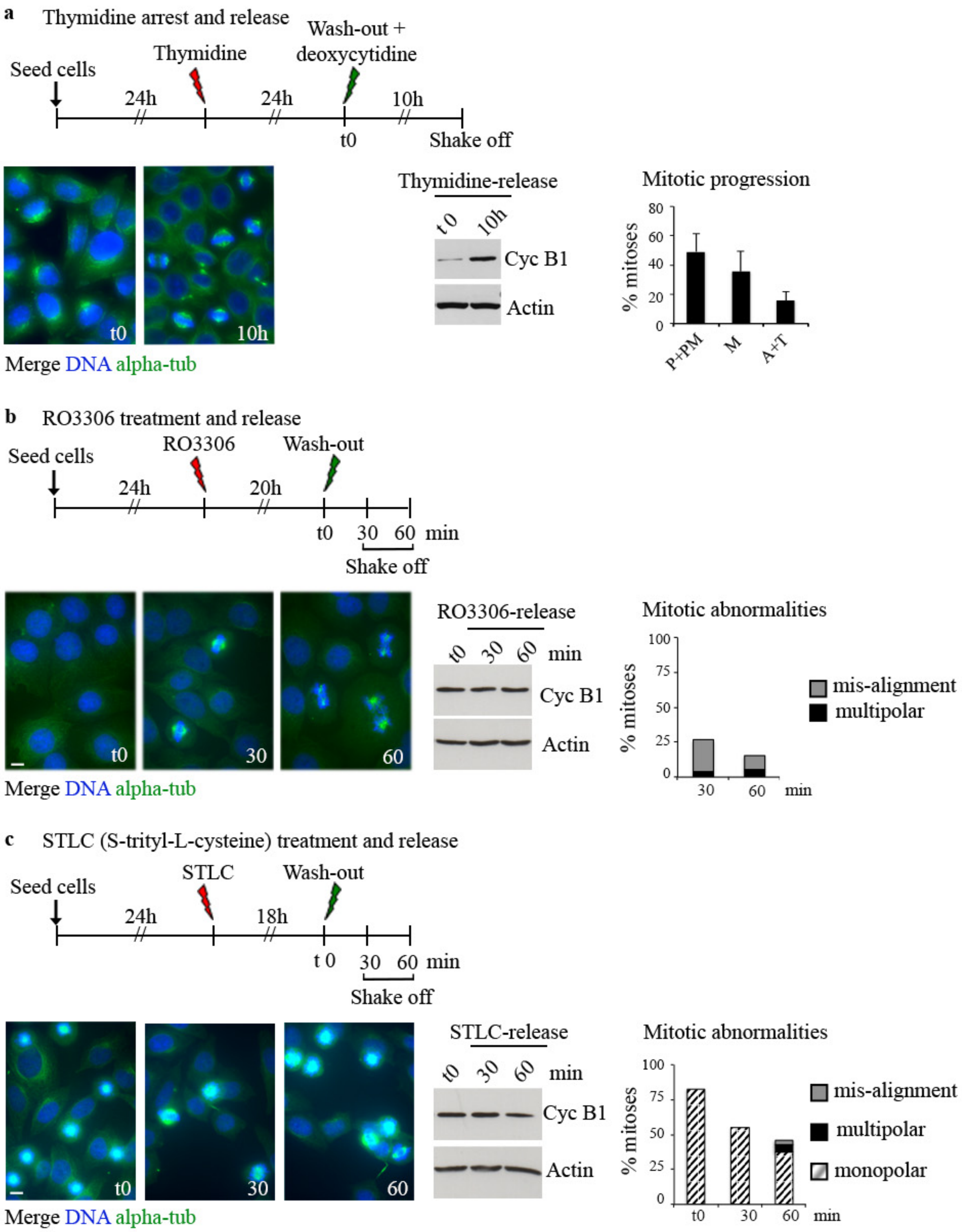

Supplementary figure 2

	Protein MW	Antibody detail	Dilution (IF and PLA)	Dilution (WB)	Produced by	Code	Immunogen
GFP	27 kDa	Rabbit polyclonal	(1:1000)	(1:500)	Abcam	ab6556	Recombinant full length protein
Imp beta	97 kDa	Mouse monoclonal	(1:2000)	(1:2000)	Abcam	ab2811	Full length native protein corresponding to Cow NTF97/Importin beta.
RAN	26 kDa	Rabbit polyclonal	(1:50)	(1:200)	Santa Cruz	SC-20802	Aminoacids 121-216 mapping at the C terminus of RAN of human origin
RANBP1	24 kDa	Rabbit polyclonal	(1:100)	(1:200)	Santa Cruz	SC-28576	Aminoacids 159-203 mapping at the C terminus of RANBP1 of mouse origin
RANBP2	358 kDa	Rabbit polyclonal	(1:2000)	(1:1000)	Santa Cruz	ab64726	Synthetic peptide (human) corresponding to 3 times repeated sequence in RANBP2 protein
NuSAP	49 kDa	Rabbit polyclonal	(1:500)	(1:1000)	Abcam	ab93779	Synthetic peptide from a region within amino acids 125 - 175 of Human NUSAP1
BUB3	37 kDa	Rabbit polyclonal	(1:250)	(1:10000)	Abcam	ab133699	A synthetic peptide corresponding to residues near the N terminus of Human Bub3

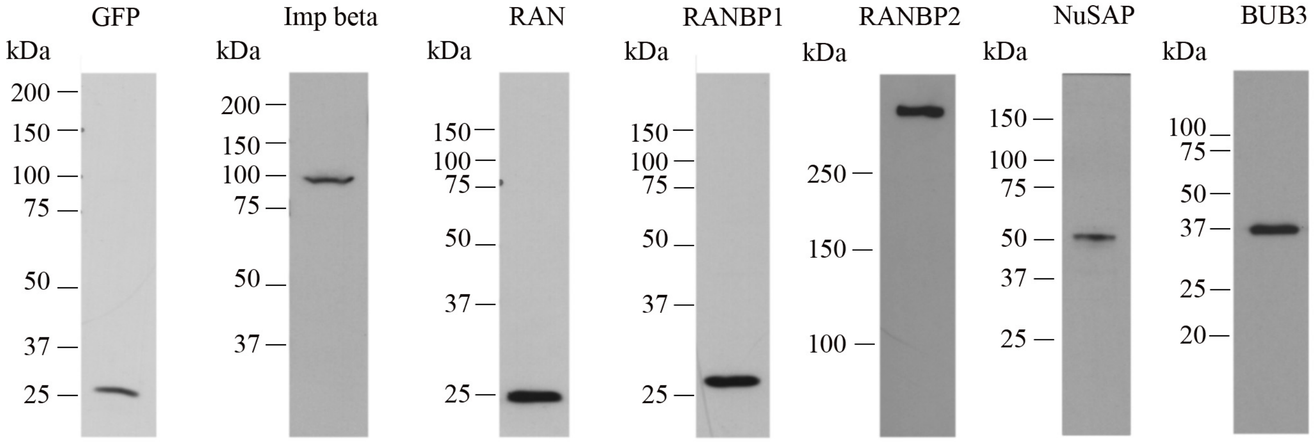

Supplementary figure 3

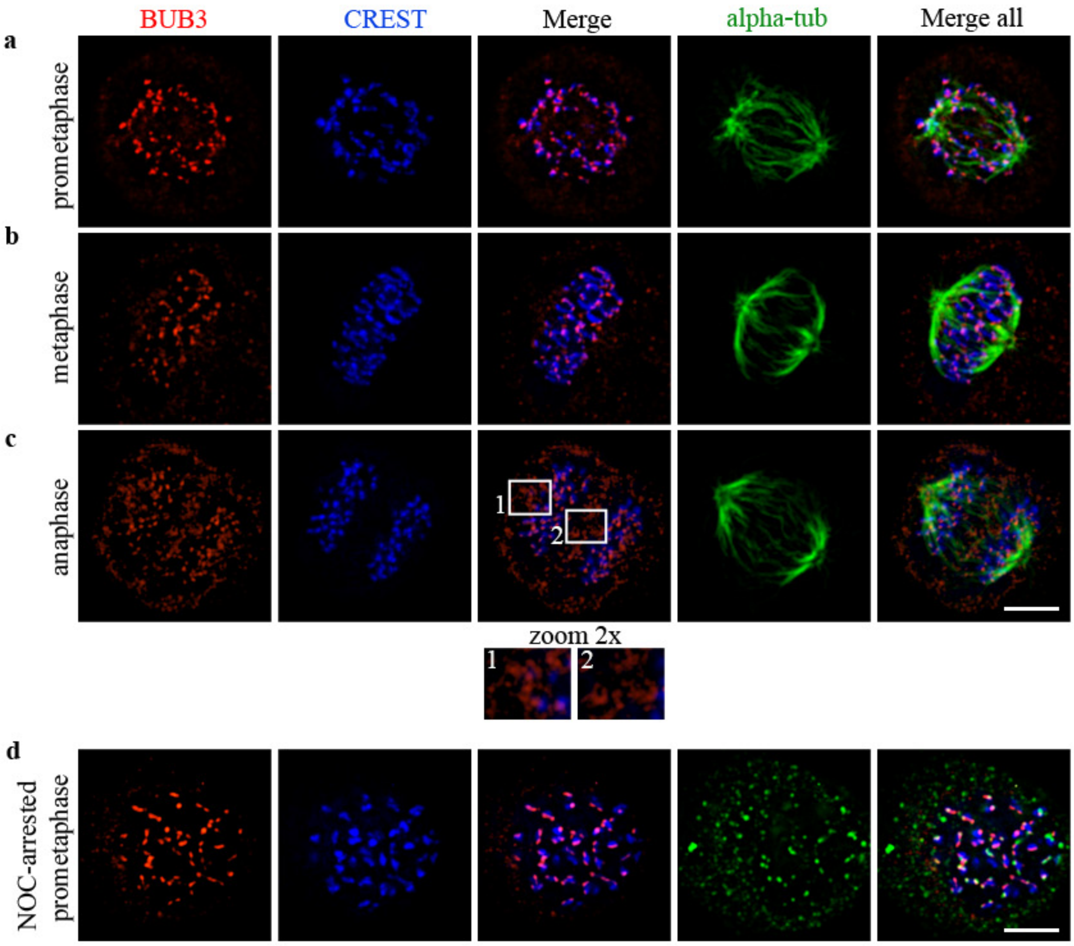

Supplementary figure 4
